# Congruency effects and individual differences in masked face recognition under limited feature visibility

**DOI:** 10.3758/s13421-025-01699-9

**Published:** 2025-03-12

**Authors:** Mengying Zhang, Melanie Sauerland, Anna Sagana

**Affiliations:** Section Forensic Psychology, Department of Clinical Psychological Science, Faculty of Psychology and Neuroscience Maastricht University, Universiteitssingel 40, PO Box 616, 6200 MD Maastricht, The Netherlands

**Keywords:** Face recognition, Congruency effect, Individual differences, Memory load

## Abstract

Recognizing masked faces is a challenge. Researchers have explored congruency-based approaches to improve face matching, with promising results. Here, we investigated whether congruency between the encoding and the retrieval conditions can improve masked face recognition when only the eyes are visible under conditions of high and low memory load. Additionally, we explored whether the advantage of congruency varied as a function of general face recognition ability. In three experiments (total *N* = 316), participants completed a face recognition task that manipulated the congruency between encoding and retrieval conditions. In congruent sets, the images featured either a full face or a partial face at encoding, paired with a full or partial face, respectively, at retrieval. In incongruent sets, the images paired a full face at encoding with a partial face at retrieval or a partial face at encoding with a full face at retrieval. The Cambridge Face Memory Test served as a measure of general face recognition ability. The results supported the hypothesis that contextual congruency improves face recognition (*η*_*p*_^*2*^* ≥* .46), and the effect remained consistent across high- and low-ability face recognition performers. Additionally, memory load shaped the confidence-accuracy relationship, such that confidence was a reliable predictor of accuracy under conditions of low (but not high) memory load. These results show that focusing on originally encoded facial features significantly improves masked target recognition, aiding law enforcement in identifying masked perpetrators and enhancing public safety.

**Supplementary information:**

The online version contains supplementary material available at 10.3758/s13421-025-01699-9.

## Introduction

Law enforcement faces a challenge when dealing with individuals who intentionally conceal their identity by wearing masks. This is because the occlusion of facial features impairs recognition (Freud et al., 2020; Guerra et al., 2022). Increasing the perceptual similarity between the compared faces – or contextual congruency – can facilitate matching and identification performance for masked faces (Estudillo & Wong, 2022; Manley et al., 2019; Sagana & Hildebrandt, 2022; Toseeb et al., 2014). Specifically, accuracy and discriminability tend to be higher when participants are asked to recognize or discriminate face pairs in congruent compared with in incongruent conditions. For example, performance improves when faces encoded wearing a headscarf, sunglasses, or a mask are subsequently presented in the same way at retrieval (Estudillo & Wong, 2022; Guerra et al., 2022; Manley et al., 2019; Noyes et al., 2021; Or et al., 2023; Toseeb et al., 2014). However, when it comes to masked perpetrators, prior research has mostly used surgical masks to occlude the faces, thereby leaving large parts of the face and several facial features visible (Carragher & Hancock, 2020; Guerra et al., 2022; Palu et al., 2023). Conversely, criminals tend to cover most of their face leaving only a few facial features visible. This raises concerns about the generalizability of earlier work on more taxing conditions. The aim of the current line of research was to test the usefulness of congruent face presentations under limited feature visibility.

The issue of limited feature visibility in relation to congruency effects in masked perpetrators is rarely addressed (see Manley et al., 2019; Sagana & Hildebrandt, 2022). The limited number of studies that utilize full face coverings are those that employ lineup procedures. These studies demonstrate that there is an advantage for congruent pairings over incongruent pairings. However, the magnitude of this effect varies depending on the target (see Sagana & Hildebrandt, 2022) and the presentation method employed (masked-masked pairings vs. masked-eyes cutout pairings; Manley et al., 2019; Sagana & Hildebrandt, 2022). A notable exception is a recent study on face-matching in which congruency effects persisted even when the masks covered most of the face (Zhang et al., 2023). These included either only showing the eyes and eyebrows (Experiment 1) or only the mouth (Experiment 2). In line with earlier work, participants were better at discriminating between congruent (e.g., masked face – masked face) than incongruent face pairs (e.g., masked/covered face – full/uncovered face) despite the heavy occlusion. However, unlike face matching, facial recognition involves perceiving facial features, but also retrieving and comparing stored representations of facial features from memory. This dual cognitive demand adds complexity, as mnemonic processes may influence recognition outcomes independently of the perceptual congruency achieved in earlier work (e.g., Estudillo & Bindemann, 2014; Guerra et al., 2022; Zhang et al., 2023). This raises concerns about the generalizability of contextual congruency effects in recognition tasks involving heavily masked targets.

Drawing on feature-based processing as our conceptual framework, we propose that the positive effects of congruency result from the forced use of feature-based processing, and these benefits should persist in challenging face recognition tasks. Specifically, if it is not possible to encode the whole face or large parts of the face, then allowing the face to be processed in its entirety during recognition can hinder performance. Feature-based processing compels the viewer to analyze specific components or features of a face, and use these for recognition, rather than relying on the overall configuration and global structure (i.e., holistically). For example, in one study, recognition of parts of the face, such as the area around the eyes, was poorer when participants viewed a full face rather than isolated features (Leder & Carbon, 2005). Feature-based processing involves a one-to-one comparison of local features (Megreya & Bindemann, 2018; Tanaka & Simonyi, 2016), forcing people to rely on the previously encoded and therefore diagnostic features. Put differently, limiting visible facial features during recognition is helpful because it increases people’s attention to the encoded features (Megreya, 2018; Megreya & Bindemann, 2009; Towler et al., 2017, 2021). Therefore, congruency effects in the context of masked faces are likely the result of the forced, situational use of feature-based processing.

The disruption of holistic processing caused by face masks also raises questions about the adaptability of individuals with superior face recognition skills to masked face recognition. Proficient face recognizers are known to process faces differently than their less-proficient counterparts (Bobak et al., 2016; Duchaine & Nakayama, 2006; Russell et al., 2009). Proficient recognizers rely more on holistic processing than other face-processing strategies (DeGutis et al., 2013; Sunday et al., 2017; Wang et al., 2012). However, as masks inhibit holistic processing (Freud et al., 2020; Guerra et al., 2022), proficient performers may increasingly rely on piecemeal (i.e., feature-by-feature) sampling techniques. For example, earlier work in face-matching has shown that while masks impair face-matching performance for all individuals, proficient performers exhibit reduced effects compared to average performers (Bennetts et al., 2022; Noyes et al., 2021). It seems that people with superior face recognition abilities make more effective use of visible features to activate their stored memory representations, regardless of the processing strategy used (Dunn et al., 2022; Royer et al., 2018). This leads to the question of whether the superior performance in proficient recognizers can be maintained in the context of masked face recognition.

Lastly, congruency of facial representations can also influence confidence ratings and decision times. Some studies suggested that congruent pairs produced more confident and faster responses than incongruent pairs (Stephens et al., 2017; Zhang et al., 2023). However, most studies either did not find the typical confidence-accuracy or decision time-accuracy relationship for masked targets (e.g., Manley et al., 2019; Sagana & Hildebrandt, 2022; Zhang et al., 2023) or, when they did, the findings were limited to encoding full faces (Hsiao et al., 2022). Additionally, face processing might be impaired under conditions of high face memory load (Cheung & Gauthier, 2010; Lamont & Stewart-Williams, 2005; Ölander et al., 2019; Podd, 1990). Given our current understanding of memory, it is unsurprising that recognition may be impaired when attempting to remember multiple faces simultaneously. Indeed, an increase in memory load (i.e., encoding 20 vs. 50 faces) can lead to a noticeable decline in recognition performance (Podd, 1990). The more objects one attempt to remember, the less precise the memory representations become, resulting in a trade-off between memory capacity and precision (Ölander et al., 2019). Consequently, the confidence-accuracy relationship may be obscured if memory load is high. The present study aims to contribute to this growing body of research by examining the role of congruency on confidence-accuracy or decision time-accuracy relationship for masked targets.

### The present work

The primary objective of the current line of research was to investigate the generalizability of congruency effects in highly restricted facial feature availability (eyes only) and high memory load (recognition task). Additionally, we explored how the congruency effect influences the confidence-accuracy and the decision time-accuracy relationship. Finally, we aimed to determine whether the congruency effect is consistent across individuals with varying levels of face recognition skills. To this end, we conducted three experiments. Experiment 1 established whether the superiority of contextual congruency extends from face matching to face recognition. Experiment 2 represents a direct replication of Experiment 1, with an emphasis on examining individual differences for masked face recognition. Experiment 3 constitutes a conceptual replication of Experiment 2 with a reduced number of study targets to reduce memory load.

In all experiments, participants completed a standard face recognition task where half of the studied faces and half of the faces to be recognized were masked. This facilitated the creation of congruent and incongruent sets. In congruent sets, images during the study and test phase were presented in similar ways, revealing the same facial features, such as the full-face (full-full condition) or a single feature face (masked-partial condition). In incongruent sets, presentation methods varied between encoding and recognition. Specifically, we presented a full-face at encoding and a partial face at retrieval and vice versa. Additionally, to assess each participant's general face recognition performance, we administered the Cambridge Face Memory Test (CFMT; Duchaine & Nakayama, 2006).

Drawing from the documented benefits of featured-based processing (Megreya, 2018; Megreya & Bindemann, 2009; Towler et al., 2017, 2021), we expected superior recognition performance in congruent (full-full; masked-partial) compared to incongruent sets (full-partial; masked–full; Hypothesis 1). Additionally, we hypothesized that people would rate confidence higher in congruent than incongruent sets (Hypothesis 2) and a stronger confidence-accuracy relationship in congruent than incongruent sets (Hypothesis 3). Likewise, we expected faster decisions for congruent than incongruent sets (Hypothesis 4) and a stronger decision time-accuracy relationship for congruent than incongruent sets (Hypothesis 5).

We also investigated the potential influence of face recognition ability on the facial congruency effect in Experiment 2 and Experiment 3. We expected face recognition ability to be positively correlated to recognition performance in both congruent and incongruent sets (Hypothesis 6). We expected high-ability face recognition performers to retain this advantage across congruent and incongruent sets. For participants with low-ability face recognition performers, we expected equal performance in congruent and incongruent sets (Hypothesis 7). Table 1 shows an overview of the hypotheses.

**Table 1:** Summary of the hypotheses and support for hypotheses across three experiments

Hypotheses		Experiment		
		E1	E2	E3
H1	Superior recognition performance in congruent sets than incongruent sets.	✓	✓	✓
H2	Higher confidence ratings in congruent than incongruent sets.	✕	✕	✓
H3	Stronger CA relationship in congruent than incongruent sets.	✕	✕	✓
H4	Faster decisions in congruent than incongruent sets.	✕	✕	✓
H5	Stronger decision time-accuracy relationship in congruent than incongruent sets.	✕	✕	✕
H6	General face recognition ability is positively correlated with recognition performance for both congruent and incongruent sets.	n/a	✓	✓
H7	High-ability face recognition performers display the congruency effect whereas low-ability face recognition performers perform equally in congruent and incongruent sets.	n/a	✕	✕

*Note.* Results consistent with the hypothesis are marked with “√” and results inconsistent with the hypothesis are marked with “×”. We tested Hypotheses 6 and 7 only in Experiments 2 and 3

## Experiment 1

The experiment received ethical approval by the Ethics Review Committee of the Faculty of Psychology and Neuroscience and it was pre-registered on the Open Science Framework (https://osf.io/cwpzh).

### Method

#### Participants

To determine the required sample sizes we used the MorePower 6.0.4 software (Campbell & Thompson, 2012). The analysis indicated that for a repeated-measures ANOVA with a within–between interaction, 128 participants were needed for Experiment 1 to detect a medium effect size (*η*_*p*_^*2*^) of .06, with *p* = .05 and power = .80. To account for potential dropouts, we aimed at testing 150 participants. Of the 197 participants who accessed the study, we excluded 61 participants with incomplete data, and 4 non-Caucasian participants to avoid the cross-race effect (Meissner & Brigham, 2001). The final sample consisted of *N* = 132 participants (94 females, 38 males, *M*_*age*_ = 24.15 years, *SD*_*age*_ = 8.37, age range: 17–63 years). Participants passed the attention check and were recruited via the research participation system of the university or the Prolific research platform. Participants gave their consent to take part in this study and received credit or 2.50 euros as compensation for their participation.

#### Design

**Fig. 1:**
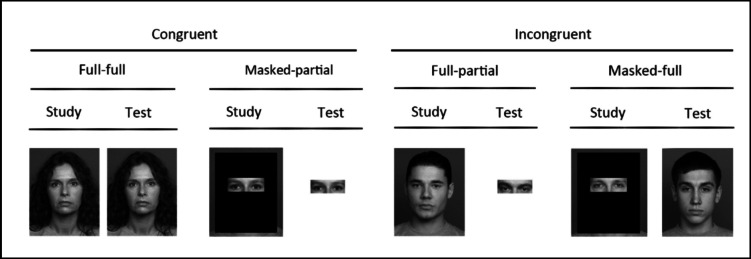
Examples of the materials used in the face recognition test in all three experiments. The congruent condition shows the same person as an example, the incongruent condition shows different people, as an example. Each participant was exposed to all conditions: full-full, masked-partial, full-partial, masked-full for face recognition

We employed a within-subjects 2 x (contextual congruency: congruent vs. incongruent) x 2 (trial type: old vs. new) design.^1^ We varied four different contextual congruency conditions within participants. The congruent sets contained (1) full faces at study and test (full-full) and (2) partial faces at study and test (masked-partial). The incongruent sets contained (3) full faces at study and partial faces at test (full-partial) and (4) masked faces at study and full faces at test (masked-full). Figure 1 illustrates all four conditions.

We measured participants’ recognition performance as the proportion of correct responses in the test. A correct response was recorded when participants identified an old face correctly as “old” and correctly identified a new face as “new”. As for signal detection theory indices (Wixted et al., 2018; Wixted & Mickes, 2014), we used the proportion of hits (correctly responding “old” when presented “old” face in the test phase) and false alarms (incorrectly responding “old” when presented “new” face in the test phase) to calculate sensitivity (*d'*) and response bias (criterion *c*).^2^ Sensitivity measures the ability to distinguish “old” face or “new” face (i.e., higher sensitivity means greater face recognition, while a zero score means chance performance). Response bias (criterion* c*) is an indicator of a bias towards declaring they recognize a face. A positive *c* value indicates a tendency to declare a “new” face (conservative bias) while a negative value indicates a tendency to declare an “old” face (liberal bias). Confidence was measured using a sliding scale from 0 (= *not confident at all)* to 100 (= *absolutely positive*). The software automatically measured the decision time in ms.

### Materials

#### Facial stimuli

The stimuli were taken from the FACES database. This database features high-resolution images of faces captured in frontal view and spanning various age groups (Ebner et al., 2010). The database contains two distinct images for each individual enabling us to use different pictures for encoding and retrieval for the same identity. All faces were depicted in grayscale, in a frontal pose, with a neutral expression and a plain background. The size of a face on the screen was 300 × 350 pixels. To create the masked conditions, we used photo-editing software (Photoshop) to superimpose a black box over the original face stimuli (see Fig. 1).

#### Procedure

This experiment was conducted online with the entire session requiring approximately 20 min. After giving consent, participants completed a typical face recognition task. The recognition task was divided into two blocks based on how faces were presented during the study phase (i.e., full faces and masked faces). Blocks were presented in random order, followed by a 20-s break. In each block, participants studied 24 faces, of which half were male and half were female. Faces were presented sequentially in the center of the screen, and each was visible for 2.5 s. Following the study phase, participants completed a 5-min filler task before the test phase began. During the test phase, participants were presented with 48 faces in each block (24 old/studied and 24 new/never seen faces). Of these 48 faces, half were presented as full faces and the other half as partial faces, with the congruency manipulations applied. The faces appeared sequentially in random order and the task was self-paced. Under each face, the participants were presented with the question “Have you ever seen this person before?” Participants determined whether the face was an “old” face (i.e., they have seen this face before) or a “new” face (i.e., they have never seen this face before) by pressing one of the two buttons (“Yes” or “No”). This display remained onscreen until the participant responded. After each trial, participants provided a confidence judgment for their responses ranging from 0 (= *not confident at all)* to 100 (= *absolutely positive*). Two attention check questions related to facial features appeared following the 10th and the 20th trials in the first block, and two more appeared following the 18th and the 32nd trials in the second block. At the end of the experiment, participants were thanked and debriefed.

### Analyses

#### Contextual congruency

To test the effects of contextual congruency on face recognition (Hypothesis 1), we pre-registered a repeated-measures ANOVA with contextual congruency as a factor. We have limited the recognition performance analyses presented to measures of signal detection (i.e., sensitivity and response bias). Recognition accuracy analyses yielded similar results and are presented in the Online Supplementary Material. Similarly, we report the preregistered exploratory analysis including participant and target gender in the Online Supplementary Material. To examine potential differences between the four conditions (full-full, masked-partial, full-partial, and masked-full) and not only as a function of congruency (congruent vs. incongruent), we also conducted paired-samples t‐tests. These analyses were not pre-registered.

#### Post-decision confidence and decision times

To explore the impact of masked face recognition on confidence and the confidence-accuracy relationship (Hypotheses 2–3) and decision time and the decision time-accuracy relationship (Hypotheses 4–5), we conducted pre-registered repeated-measures ANOVAs for confidence and decision time (decision time was normalized using a log-10 transformation). We included contextual congruency, accuracy as factors along with their interaction term in the analysis. As for paired t-tests, we only report the masked face comparison (masked-partial vs. masked-full) in the main text. Other comparisons can be found in the Online Supplementary Material. In addition to the ANOVAs, to deepen our understanding of the relationship between confidence and accuracy, we also conducted ROC analysis and CAC analysis. These results can also be found in the Online Supplementary Material.

### Results

#### Sensitivity and response bias

Consistent with our expectations (Hypothesis 1), the repeated-measures ANOVA on sensitivity, with contextual congruency as a factor showed a significant main effect of congruency, *F*(1, 131) = 224.98, *p* < .001, *η*_*p*_^*2*^ = .63, with a large effect size. Specifically, participants showed a higher sensitivity for congruent (*M* = 1.12, *SE* = 0.06) than incongruent sets (*M* = 0.32, *SE* = 0.04). Additionally, participants showed higher sensitivity in the full-full condition than in any other condition, all *ts*(131) *≥* 11.17, *p*s < .001, *d*s* ≥* 1.24. The masked-partial condition outperformed the full-partial condition, *t*(131) = 2.95, *p* = .004, *d* = 0.34, and the masked-full condition, *t*(131) = 4.91, *p* < .001, *d* = 0.53. Sensitivity between the two incongruent conditions (full-partial vs. masked-full) did not differ, *t*(131) = −1.79, *p* = .076, *d* = 0.20. Table 2 summarizes the mean sensitivity for all conditions.

**Table 2:** Descriptive statistics for sensitivity (*d’*) and response bias (*c*) for congruent and incongruent sets in three experiments

		Sensitivity (*d*^*’*^)				Response bias (*c*)			
		Congruent		Incongruent		Congruent		Incongruent	
		Full-full^a^	Masked-partial^b^	Full-partial^c^	Masked-full^d^	Full-full^a^	Masked-partial^b^	Full-partial^c^	Masked-full^d^
Experiment 1	*M (SD)*	1.66 (1.04)	0.59 (0.62)^a***^	0.25 (0.66)^ab***^	0.38 (0.62)^a***b**^	0.16 (0.58)	−0.04 (0.61)^a***^	0.10 (0.64)^b**^	0.32 (0.53)^a**bc***^
	95% CI	[1.51, 1.81]	[0.50, 0.68]	[0.16, 0.35]	[0.29, 0.47]	[ 0.08, 0.24]	[−0.13, 0.05]	[0.01, 0.19]	[0.24, 0.40]
Experiment 2	*M (SD)*	1.48 (0.97)	0.56 (0.61)^a***^	0.23 (0.69)^ab***^	0.39 (0.62)^a***bc*^	0.09 (0.65)	−0.13 (0.67)^a**^	0.06 (0.73)^b*^	0.48 (0.66)^abc***^
	95% CI	[1.33, 1.63]	[0.47, 0.65]	[0.12, 0.34]	[0.29, 0.49]	[−0.01, 0.19]	[−0.23, −0.03]	[−0.05, 0.17]	[0.38, 0.58]
Experiment 3	*M (SD)*	1.86 (0.96)	0.91 (0.95)^a***^	0.43 (0.84)^a***b**^	0.61 (1.02)^a***^	0.03 (0.46)	0.00 (0.65)	0.06 (0.66)	0.41 (0.66)^abc***^
	95% CI	[1.67, 2.05]	[0.72, 1.10]	[0.26, 0.60]	[0.41, 0.82]	[−0.06, 0.12]	[−0.13, 0.13]	[−0.07, 0.19]	[0.28, 0.54]

*Note*. The superscript letters indicate significant differences from ^a^ = full-full, ^b^ = masked-partial, ^c^ = full-partial, ^d^ = masked-full, at ^*^p < .05, ^**^p < .01, ^***^p < .001. The mean sensitivity and response bias values are calculated using the 1/(2*N*) correction

A repeated-measures ANOVA on response bias, with contextual congruency as a factor, revealed a significant main effect of congruency, *F*(1, 113) = 24.83, *p* < .001, *η*_*p*_^*2*^ = .18. Participants displayed more conservative response bias in incongruent sets (*M* = 0.18, *SE* = 0.05) than in congruent sets (*M* = −0.02, *SE* = 0.05). Pairwise comparisons revealed differently between masked conditions (cf. Table 2), showing that the masked-full condition elicited more conservative responses than other conditions, *ts*(131) *≥* −2.85, *p*s ≤ .005, *d*s *≥* 0.29. However, response bias was more liberal in the masked-partial condition than in the full-full condition, *t*(131) = 3.46, *p* < .001, *d* = 0.34, and full-partial condition, *t*(131) = 3.12, *p* = .002, *d* = 0.22. Notably, an unbiased response was only observed in the full-partial condition, *t*(131) = 1.86, *p* = .065, *d* = 0.16. These findings underscore the different criteria for recognizing masked faces, showing participants displayed a more conservative bias in incongruent masked conditions.

#### Post-decision confidence

We then conducted a repeated-measures ANOVA on confidence, with contextual congruency and accuracy as factors. Inconsistent with Hypothesis 2 and 3, neither congruency, *F*(1, 131) = 1.50, *p* = .223, *η*_*p*_^*2*^ = .01, nor accuracy , *F*(1, 131) = 3.53, *p* = .063, *η*_*p*_^*2*^ = .03, had an effect on confidence. The interaction between congruency and accuracy was also not significant, *F*(1, 131) = 0.96, *p* = .330, *η*_*p*_^*2*^ < .01. No systematic differences emerged between the masked-partial condition and the masked-full condition (cf. Table 3 and Online Supplementary Material).

**Table 3:** Descriptive statistics for mean confidence and mean decision time for congruent and incongruent sets in three experiments

		Confidence				Decision time			
		Congruent		Incongruent		Congruent		Incongruent	
		Full-full^a^	Masked-partial^b^	Full-partial^c^	Masked-full^d^	Full-full^a^	Masked-partial^b^	Full-partial^c^	Masked-full^d^
Experiment 1	*M (SD)*	59.53 (15.99)	53.88 (16.71)^a***^	59.29 (16.49)^b***^	54.14 (16.84)^ac***^	2.82 (1.51)	3.16 (1.58)^a***^	2.82 (1.51)^b***^	3.16 (1.58)^ac***^
	95% CI	[57.23, 61.83]	[51.48, 56.28]	[56.92, 61.66]	[51.72, 56.56]	[2.63, 3.02]	[3.02, 3.29]	[2.63, 2.95]	[2.95, 3.29]
Experiment 2	*M (SD)*	59.80 (16.78)	56.18 (18.34)^a***^	59.65 (17.04)^b***^	56.23 (17.96)^ac***^	2.75 (1.51)	3.24 (1.66)^a***^	2.75 (1.45)^b***^	3.16 (1.62)^ac***^
	95% CI	[57.21, 62.39]	[53.27, 59.09]	[57.02, 62.28]	[53.46, 59.01]	[2.57, 2.95]	[3.02, 3.47]	[2.63, 2.95]	[2.95, 3.29]
Experiment 3	*M (SD)*	73.14 (14.03)	57.30 (16.64)^a***^	47.46 (18.16)^ab***^	53.19 (18.12)^ac***b**^	2.82 (1.58)	2.69 (1.48)	2.88 (1.55)	3.55 (1.66)^abc***^
	95% CI	[70.37, 75.91]	[54.02, 60.58]	[43.88, 51.04]	[49.62, 56.76]	[2.57, 3.09]	[2.51, 2.88]	[2.51, 3.16]	[3.24, 3.89]

*Note.* The superscript letters indicate significant differences from ^a^ = full-full, ^b^ = masked-partial, ^c^ = full-partial, ^d^ = masked-full, at ^*^p < .05, ^**^p < .01, ^***^p < .001

#### Decision time

Similar to confidence, the repeated-measures ANOVA on decision time, with contextual congruency and accuracy as factors, yielded no significant results. Contrary to Hypotheses 4 and 5, there was no main effect of contextual congruency on decision time, *F*(1, 131) = 1.71, *p* = .194, *η*_*p*_^*2*^ = .01, and no main effect of accuracy on decision times, *F*(1, 131) = 0.33, *p* = .568, *η*_*p*_^*2*^ < .01. The interaction between congruency and accuracy was also not significant, *F*(1, 131) = 0.04, *p* = .851, *η*_*p*_^*2*^ < .01. In comparison to the masked-face condition, participants made faster decisions in the full-face condition, but no other systematic differences emerged (cf. Table 3 and Online Supplementary Material).

### Discussion

The results of Experiment 1 indicated that congruency between the encoding and retrieval phase improved face recognition. Participants showed higher sensitivity in congruent (full-full; masked-partial) compared to incongruent face sets (full-partial; masked-full). This is consistent with previous studies (Estudillo & Wong, 2022; Guerra et al., 2022; Or et al., 2023). The large effect size underscores the substantial impact of congruency on recognition performance. Additionally, the masked-partial condition also showed better performance than the masked-full condition, highlighting the critical role of congruency in facilitating recognition of masked targets. For response bias, participants adopted a more conservative approach in incongruent sets compared to congruent sets. This finding indicates that participants were more cautious when making recognition under incongruent conditions, possibly due to the increased difficulty in matching encoded and retrieved information.

In terms of markers of accuracy, contrary to our hypotheses, we did not observe significant main effects of congruency or accuracy on post-decision confidence and decision time, nor an interaction between these factors. The lack of significant relationships between accuracy and confidence and decision time may be attributed to several factors. One possibility is that the overall low performance in Experiment 1 obscured potential relationships. Another explanation could be that the specific sample characteristics influenced these outcomes. To rule out the possibility that these outcomes were due to sample-specific characteristics, we conducted a direct replication and extension in Experiment 2. Additionally, Experiment 2 examined individual variability in masked recognition performance.

## Experiment 2

The experiment received ethical approval by the Ethics Review Committee of the Faculty of Psychology and Neuroscience.

### Method

#### Participants

The same power analysis as Experiment 1 indicates that we need 128 participants to detect a medium effect size (*η*_*p*_^*2*^) of .06, with *p* = .05 and power = .80. Of the 184 participants who accessed the study, we excluded 68 participants with incomplete data and two participants who took over 2 h to complete the task. The final sample consisted of *N* = 114 participants (57 females, 57 males, *M*_*age*_ = 32.73 years, *SD*_*age*_ = 12.99, age range: 19–70 years).

#### Design and materials

The design and materials were similar to Experiment 1. The only difference was the inclusion of the Cambridge Face Memory Test (CFMT; Duchaine & Nakayama, 2006) after the recognition task. The CFMT was used to test participants’ face recognition ability, which includes three progressively challenging blocks. In the first block, participants memorize three pictures (a left one-third profile, a frontal view, and a right one-third profile) and then select the face they had previously viewed from a set of three choices. In the second block, participants memorize six target faces and identify novel faces among them. The third block mirrors the second but introduces visual noise to the test images. There is a total of 72 possible points on the test. The highest value with the sum of the correct responses is 72. To quantify face recognition ability, we employed a straightforward performance percentage calculation (CFMT performance = (Number of correct responses / Total number of items) ×100), this calculation yields a score ranging from 0 to 1, where 1 represents perfect performance in CFMT.

#### Procedure

This experiment was conducted online with the entire session requiring approximately 35 min. The procedure for the face recognition task was identical to Experiment 1. The only difference was that participants performed the CFMT after the face recognition task in Experiment 2. Participants were also thanked and debriefed at the end of the experiment.

#### Analyses

The analysis of the congruency effect on face recognition, post-decision confidence, and decision times were the same as in Experiment 1. Furthermore, to investigate the effect of general recognition ability (CFMT performance) on face recognition performance for masked targets under different contextual congruency conditions (Hypothesis 6), we performed a series of correlational analyses. The analyses included general recognition ability and recognition performance for each congruency condition.

To explore different performance patterns, we analyzed the performance of participants who were in the top 5% (high-ability performers) and the bottom 5% (low-ability performers; for a similar approach, see Estudillo et al., 2021; Fysh et al., 2020). These participants were selected based on their rank sum across the CFMT score. To test the effects of contextual congruency between high- and low-ability performers on face recognition (Hypothesis 7), we conducted repeated-measures of ANOVA with contextual congruency and CFMT performance (high vs. low) and their interaction effect as factors. Lastly, to examine potential differences between high-ability performers and low-ability performers in four conditions (full-full, masked-partial, full-partial, and masked-full), we used paired samples t-tests. Finally, given the small sample sizes of high- and low-ability performers, we extended the original 5% cut-offs to include the top and bottom quartiles and incorporated Bayes factors into the ANOVAs. These adjustments were made to enhance our understanding of the effect, leading to more robust and reliable conclusions.

### Results

#### Sensitivity and response bias

To test the effect of congruency on sensitivity we conducted a repeated-measure ANOVA with contextual congruency as a factor. In line with Hypothesis 1 and Experiment 1, the main effect of the congruency on sensitivity was significant, *F*(1, 113) = 115.65, *p* < .001, *η*_*p*_^*2*^ = .51, with a large effect size. Participants showed a higher sensitivity for congruent (*M* = 1.02, *SE* = 0.06) than incongruent sets (*M* = 0.31, *SE* = 0.05). In particular, participants showed higher sensitivity in the full-full condition than in any of the other conditions (cf. Table 2), *t*s(113) *≥* 9.65, *p*s < .001, *d*s *≥* 1.14. The masked-partial condition outperformed the full-partial condition, *t*(113) = 2.27, *p* = .025, *d* = 0.28, and the masked-full condition, *t*(113) = 3.94, *p* < .001, *d* = 0.51. Moreover, unlike Experiment 1, sensitivity between the two incongruent conditions (full-partial vs. masked-full) differed significantly, *t*(113) = −2.07, *p* = .041, *d* = 0.24.

A repeated-measures ANOVA on response bias, with contextual congruency as a factor, showed a significant main effect of congruency, *F*(1, 113) = 24.83, *p* < .001, *η*_*p*_^*2*^ = .18. Participants displayed more conservative response bias in incongruent sets (*M* = 0.18, *SE* = 0.05) than in congruent sets (*M* = −0.02, *SE* = 0.05). Pairwise comparisons showed differences between masked conditions (cf. Table 2). The masked-full condition elicited more conservative responses than the other conditions, *t*s(113) *≥* −4.98, *p*s < .001, *d*s *≥* 0.60. However, response bias was more liberal in the masked-partial condition than in the full-full condition, *t*(113) = 3.21, *p* = .002, *d* = 0.33, and full-partial condition, *t*(113) = 2.59, *p* = .011, *d* = 0.27. Additionally, participants displayed unbiased responses when encoding a full face, regardless of whether a full face or a partial face was presented during the retrieval phase, *t*s(113) ≤ 1.49, *p*s ≥ .139, *d*s ≤ 0.14. These findings were consistent with Experiment 1, and once again underscored the robust influence of contextual congruency on recognition performance.

#### Post-decision confidence

Inconsistent with Hypotheses 2 and 3, the ANOVA on confidence, with contextual congruency and accuracy as factors, returned null findings. There was no significant main effect of congruency, *F*(1, 113) < .01, *p* = .964, *η*_*p*_^*2*^ < .01, and no main effect of accuracy on confidence, *F*(1, 113) = 2.02, *p* = .158, *η*_*p*_^*2*^ = .02. The interaction between congruency and accuracy was also not significant, *F*(1, 113) = 0.63, *p* = .430, *η*_*p*_^*2*^ = .01. The results were consistent with Experiment 1, showing no systematic differences between the masked-partial and the masked-full condition (cf. Table 3 and Online Supplementary Material).

#### Decision time

Analogous to Experiment 1, the ANOVA on decision time, with contextual congruency and accuracy as factors returned null findings. Contrary to Hypotheses 4 and 5, neither contextual congruency, *F*(1, 113) = 0.98, *p* = .324, *η*_*p*_^*2*^ = .01, nor accuracy, *F*(1, 113) = 0.85, *p* = .359, *η*_*p*_^*2*^ = .01, significantly affected decision time. The interaction between congruency and accuracy was also not significant, *F*(1, 113) = 0.38, *p* = .537, *η*_*p*_^*2*^ < .01. Finally, no significant differences between the masked-partial condition and the masked-full condition emerged (cf. Table 3 and Online Supplementary Material).

#### Individual differences in masked face recognition

**Fig. 2:**
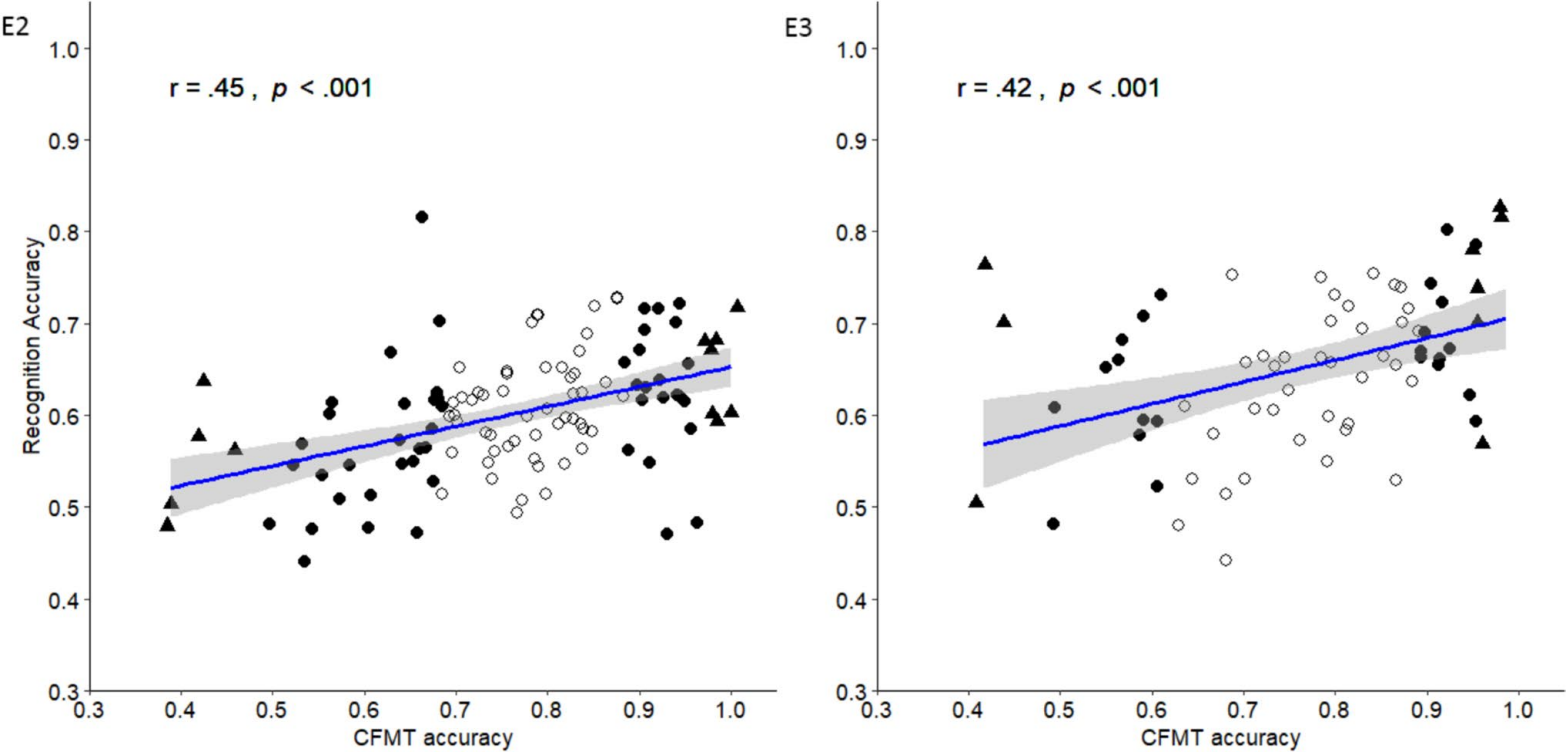
Correlations between face recognition performance and the Cambridge Face Memory Test (CFMT) in Experiment 2 (**E2**) and Experiment 3 (**E3**)*.* Participants with top 5% and bottom 5% scores in CFMT are presented by triangles; the top 25% and the bottom 25% performers are presented by solid circles. 95% CI in gray. The individual dots represent the participants in the study

To explore the relationship between participants’ general recognition ability and recognition performance for masked targets, we first examined the correlation between participants’ CFMT performance and overall recognition accuracy (see Fig. 2, E2). The mean CFMT performance was 76.45% (*SD* = 14.42, range: 39–100%). This translates to a CFMT score of *M* = 55.04 (*SD* = 10.38, range: 28–72).

**Fig. 3:**
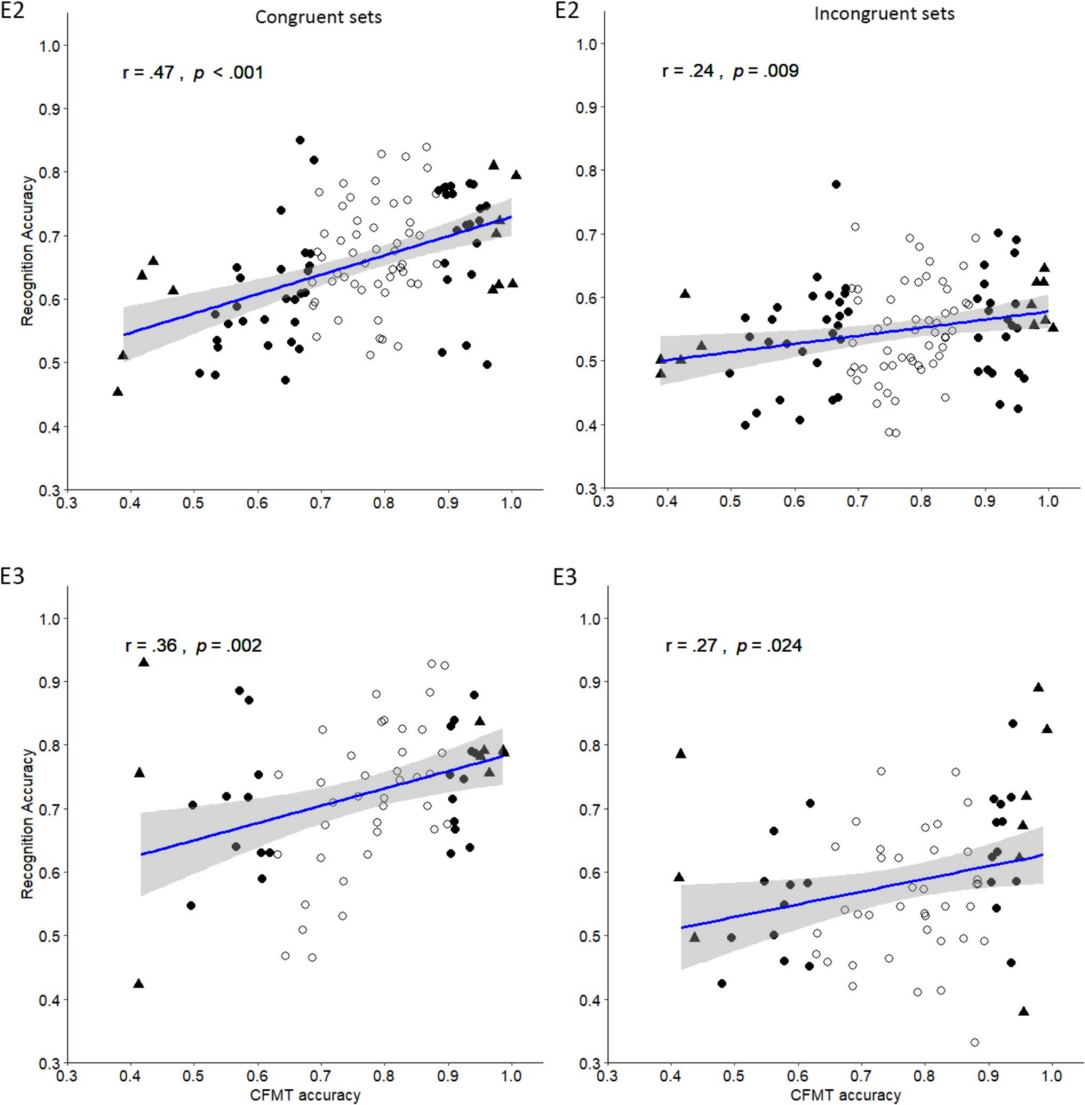
Correlations between Face Recognition Performance and the Cambridge Face Memory Test (CFMT) for Congruent (**left**) and Incongruent Sets (**right**) in Experiment 2 (**E2**) and Experiment 3 (**E3**)*.* Scatterplots show the relationship between the CFMT and face recognition performance for congruent and incongruent face sets. Participants with the top 5% and bottom 5% CFMT scores are represented by triangles; participants with the top 25% and the bottom 25% cutoff are presented by solid circles, 95% CI in gray. The individual dots represent the participants in the study

The correlation between CFMT performance and masked face recognition accuracy was moderate and significant, *r*(114) = .45, *p* < .001, 95% CI [.28, .60]. Then, we explored the effect of contextual congruency on the relationship between general recognition ability and recognition accuracy for masked targets (Fig. 3, E2 left). In the congruent sets, the correlation between CFMT performance and recognition accuracy was significant and moderate, *r*(114) = .47, *p* < .001, 95% CI [.28, .60]. However, for the incongruent sets (Fig. 3, E2 right), the correlation between CFMT performance and recognition accuracy was significant but small, *r*(114) = .24, *p* = .009, 95% CI [.06, .41]. Lastly, for all but the full-partial condition the correlations between CFMT performance and each face condition were significant and positive (cf. Online Supplementary Material). The results are largely consistent with Hypothesis 6. However, the drop in strength for the incongruent sets is noteworthy.

#### Differences between high- and low-ability performers

**Fig. 4:**
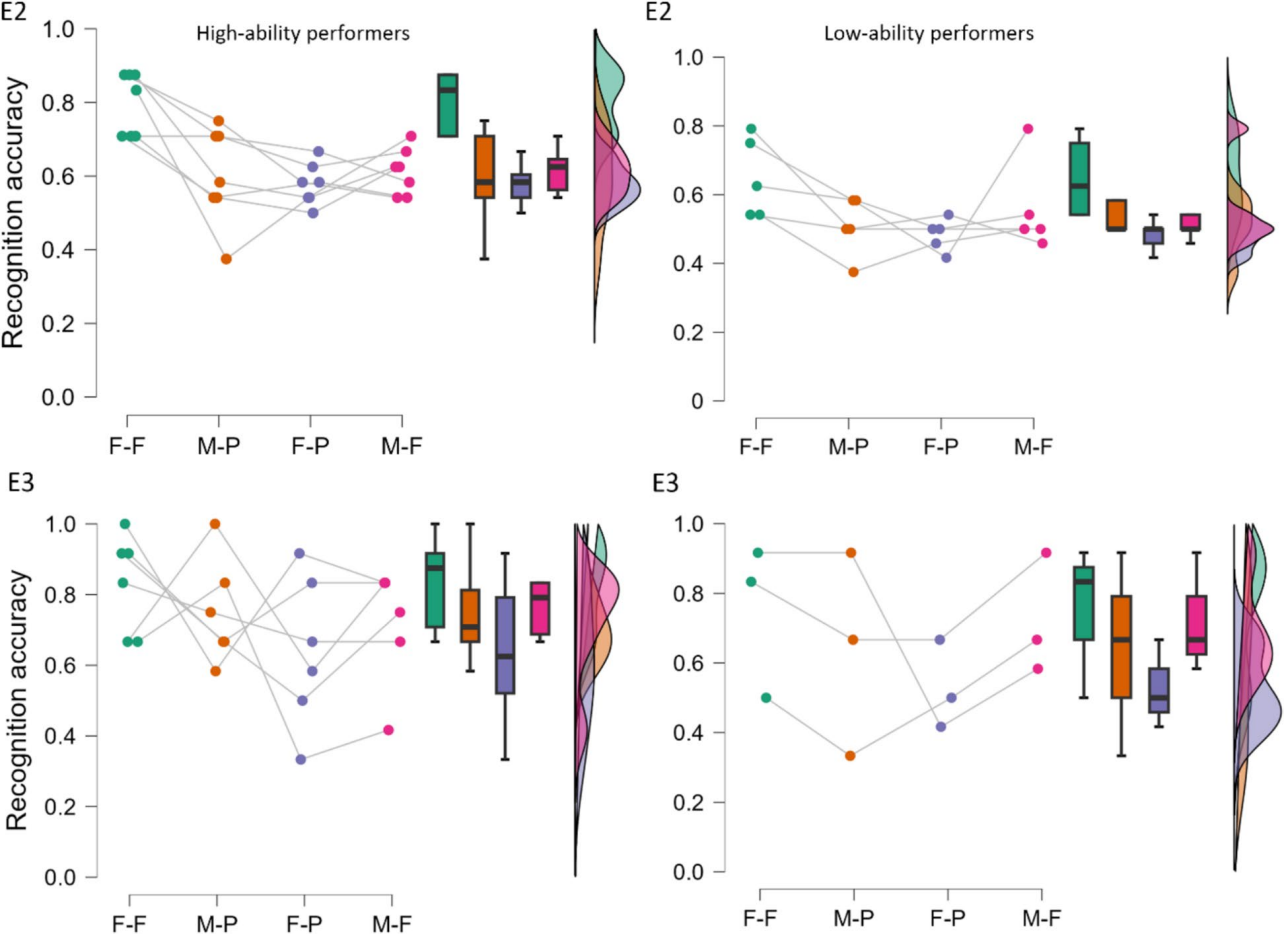
Face recognition performance of high-ability performers (top 5% performers, **left**) and low-ability performers (bottom 5% performers, **right**) in each condition in Experiment 2 (**E2**) and Experiment 3 (**E3**). Raincloud plots show the proportion correct responses in the face recognition task for the top 5% of the Cambridge Face Memory Test (CFMT) scores group (**left**) and bottom 5% of CFMT scores group (**right**) in each face condition. F-F: full-full condition, M-P: masked-partial condition, F-P: full-partial condition, M-F: masked-full condition. Colored points show the performance of each participant in each condition; box plots display the median and upper/lower quartiles for each group in each condition, and histograms represent the distribution of data in each condition

We then turned to examining differences between high and low ability face recognition performers in congruent and incongruent sets (see Fig. 4). The mean performance of high-ability performers (*n* = 7) was *M* = 98.41% *(SD* = 1.25%, range: 97–100%). This translates to a CFMT score of *M* = 70.86 (*SD* = 0.90, range: 70–72). The mean performance of low-ability performers (*n* = 5) was *M* = 41.67% (*SD* = 2.95%, range: 39–46%). This translates to a CFMT score of *M* = 30 (*SD* = 2.12, range: 28–33).

To test for differences, we ran a repeated-measures ANOVA on accuracy, with contextual congruency as a factor. Given the small sample size and to address the potential limitations of relying solely on* p*-values in such cases, we supplemented the analyses with the inclusion of a Bayesian ANOVA. Next to the established main effect of contextual congruency on recognition accuracy, *F*(1, 10) = 15.46, *p* = .003, *η*_*p*_^*2*^ = .61, BF_10_ = 25.34, we found a significant main effect of CFMT performance on recognition accuracy,* F*(1, 10) = 8.44, *p* = .016, *η*_*p*_^*2*^ = .46, BF_10_ = 2.43. While the *p* value and the large effect size indicate that high-ability performers (*M* = 64.70%, *SE* = 2.20%) were more accurate than low-ability performers (*M* = 55.00%, *SE* = 2.60%), the small Bayes factor suggests that this finding should be interpreted with caution. Lastly, the interaction effect between contextual congruency and performance was not significant, *F*(1, 10) = 1.23, *p* = .293, *η*_*p*_^*2*^ = .11, BF_10_ = 0.69.

**Fig. 5:**
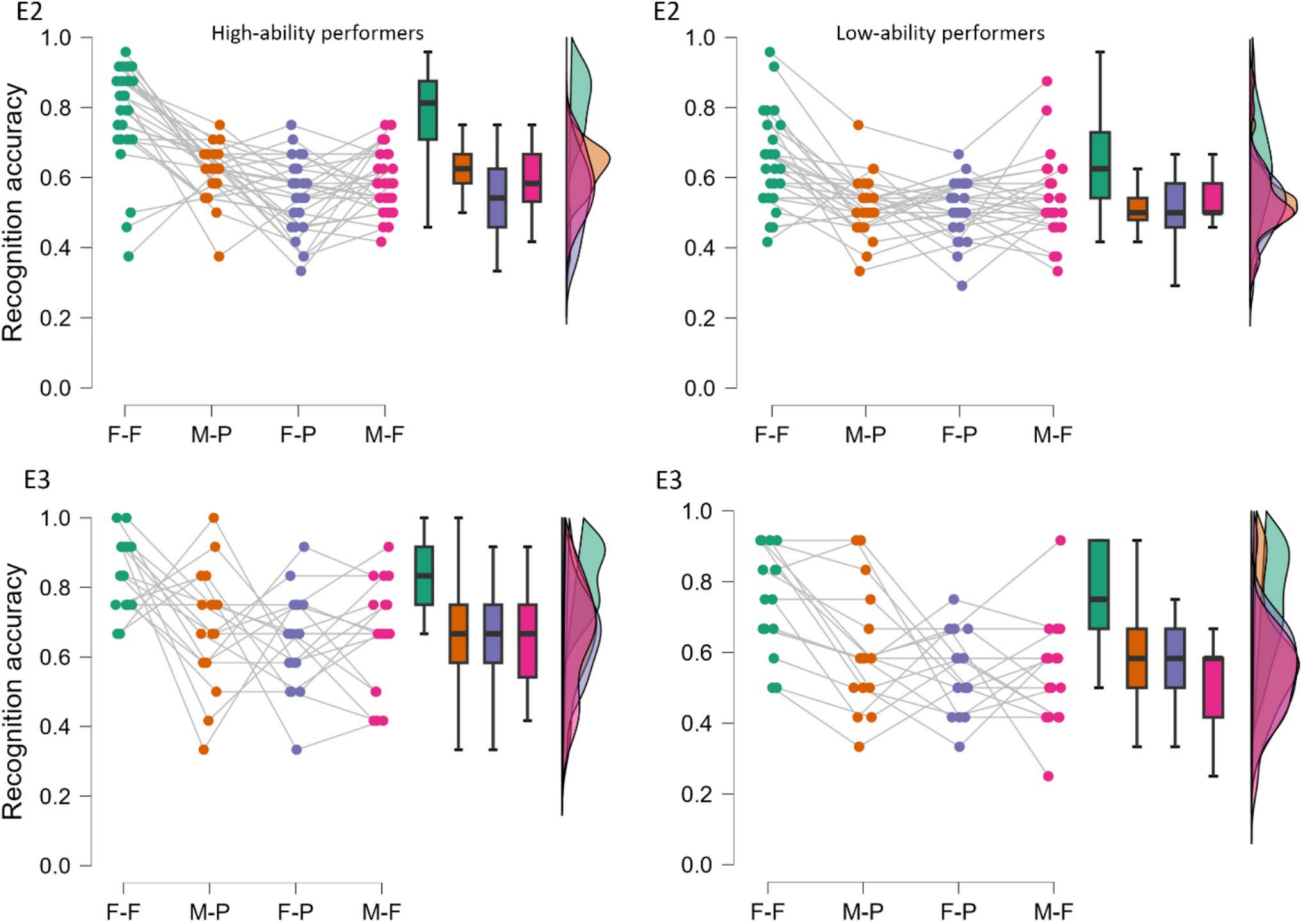
Face recognition performance of high-ability (top 25% performers, **left**) and low-ability performers (bottom 25% performers, **right**) in each condition in Experiment 2 (**E2**) and Experiment 3 (**E3**). Raincloud plots show the proportion correct responses in the face recognition task for the top 25% of the Cambridge Face Memory Test (CFMT) scores group (**left**) and bottom 25% of the CFMT scores group (**right**) in each face condition. F-F: full-full condition, M-P: masked-partial condition, F-P: full-partial condition, M-F: masked-full condition. Colored points show the performance of each participant in each condition; box plots display the median and upper/ lower quartiles for each group in each condition, and histograms represent the distribution of data in each condition

To further address power concerns given the small sample size, we extended our analysis by broadening the margins to the top and bottom 25% performance rate (see Fig. 5). The mean performance of the top 25% performers (*n* = 28) was *M* = 93.80% (*SD* = 3.45%, range: 89–100%). This translates to a CFMT score of *M* = 67.54 (*SD* = 2.49, range: 64–72). The mean performance of the bottom 25% performers (*n* = 27) was *M* = 56.74% (*SD* = 8.94%, range 39–67%). This translates to a CFMT score of* M* = 40.85 (*SD* = 6.44, range: 28–48). This analysis once again showed the significant main effect of contextual congruency, *F*(1, 53) = 68.65, *p* < .001, *η*_*p*_^*2*^ = .56, BF_10_ = 3.85 × 10^7^, and CFMT performance on recognition accuracy,* F*(1, 53) = 17.79, *p* < .001, *η*_*p*_^*2*^ = .25, BF_10_ = 98.15. Importantly, these main effects were qualified by the significant interaction effect between contextual congruency and CFMT performance, *F*(1, 53) = 11.09, *p* = .002, *η*_*p*_^*2*^ = .17, BF_10_ = 19.83. The Bayes factor supported the frequentist analysis suggesting a strong interaction effect. Simple main-effects analysis showed that for high-ability performers, accuracy was higher in congruent (*M* = 70.00%, *SE* = 1.70%) than incongruent sets (*M* = 56.60%, *SE* = 1.40%), *F*(1, 27) = 50.32, *p* < .001, *η*_*p*_^*2*^ = .65. Low-ability performers were also more accurate in congruent (*M* = 58.20%, *SE* = 1.60%) than incongruent sets (*M* = 52.50%, *SE* = 1.60%), *F*(1, 26) = 16.34, *p* < .001, *η*_*p*_^*2*^ = .43, but the effect was smaller compared to high-ability performers.

Taken together, these findings indicate that contextual congruency had a pronounced positive impact on face recognition performance, regardless of the participants' general face recognition ability (CFMT performance). However, the difference in recognition performance between congruent and incongruent sets was more pronounced in high-ability performers than in low-ability performers.

### Discussion

The results of Experiment 2 robustly replicated and extended the findings from Experiment 1, underscoring the critical role of congruency in enhancing face recognition performance when targets are masked. Similar to Experiment 1, participants adopted a more conservative response bias in incongruent sets compared to congruent sets. Furthermore, general face recognition ability was positively correlated with recognition accuracy in both congruent and incongruent conditions. This effect was more pronounced among high-ability performers. Low-ability performers also demonstrated a congruency effect, albeit weaker. This discrepancy aligns with prior research, suggesting individuals with stronger general face recognition abilities tend to perform better even under restricted or masked recognition conditions (Bennetts et al., 2022; Noyes et al., 2021).

However, overall recognition performance remained rather poor, possibly limiting our ability to detect the effects of congruency or accuracy on post-decision confidence and decision time. Given that increases in memory load can lead to a noticeable decline in recognition performance (Cheung & Gauthier, 2010; Podd, 1990), it is likely that the memory load negatively affected participants' performance. To address this potential limitation, Experiment 3 serves as a conceptual replication of Experiment 2, with a reduced number of study targets to account for the potential impact of cognitive memory overload on the observed results. By reducing the memory load, we aim to enhance overall recognition performance and provide a more sensitive test of the hypothesized relationships between congruency, accuracy, confidence, and decision time.

## Experiment 3

The experiment received ethical approval by the Ethics Review Committee of the Faculty of Psychology and Neuroscience and it was pre-registered on the Open Science Framework (https://osf.io/6zbg4).

### Method

#### Participants

For Experiment 3, we based our power calculation (*η*_*p*_^*2*^ = .54) on the size of the congruency effect observed in Experiment 2. While this effect size in Experiment 2 indicated that we had sufficient power to detect significant effects, we noticed that the sample size was relatively small. To ensure the robustness of our findings, we also conducted a statistical analysis aimed at detecting a medium effect size *f* = .25, with *p* = .05 and power = .95 for a repeated-measures ANOVA with a within–between interaction. Our analysis indicated that we needed a minimum of 54 participants. To account for potential dropouts, we aimed at testing 70 participants. Of the 82 participants who accessed the study, we excluded 12 participants with incomplete data. The final sample consisted of 70 participants (35 females, 35 males, *M*_*age*_ = 31.53 years, *SD*_*age*_ = 9.14, age range: 19–63 years).

#### Design and materials

The design and materials were the same as in Experiment 2.

#### Procedure

This experiment was conducted online with the entire session requiring approximately 30 min. Experiment 3 followed the same procedure as Experiment 2 except that the number of targets in the recognition task was lower to reduce the memory load. Specifically, participants studied 12 (instead of 24) faces in the encoding phase and at the retrieval phase, they were presented with 24 (instead of 48) faces for recognition.

#### Analyses

All analyses were pre-registered. The analysis of the congruency effect, the post-decision confidence^3^ and decision times as well as the comparisons of the four face conditions (full-full; masked-partial; full-partial; masked-full) were the same as in Experiments 1 and  2. The analyses of individual differences were the same as the Experiment 2.

### Results

#### Sensitivity and response bias

The ANOVA on sensitivity, with contextual congruency as a factor, revealed a significant main effect of congruency with a large effect size, *F*(1, 69) = 58.45, *p* < .001, *η*_*p*_^*2*^ = .46. Participants showed a higher sensitivity for congruent (*M* = 1.38, *SE* = 0.09) than incongruent sets (*M* = 0.52, *SE* = 0.08). Participants showed higher sensitivity in the full-full condition than in any other condition (see Table 2), *t*s(69) *≥* 6.64, *p*s < .001, *d*s* ≥* 0.99. The masked-partial condition outperformed the full-partial condition, *t*(69) = −2.89, *p* = .005, *d* = 0.54, but not the masked-full condition, *t*(69) = 1.94, *p* = .057, *d* = 0.30, as in Experiments 1 and 2. Additionally, the sensitivity did not differ between the full-partial condition and the masked-full condition, *t*(69) = −1.23, *p* = .223, *d* = 0.19.

The repeated-measures ANOVA on response bias, with contextual congruency as factor showed once again a significant main effect of congruency, *F*(1, 69) = 15.65, *p* < .001, *η*_*p*_^*2*^ = .19. Participants were more conservative in incongruent (*M* = 0.23, *SE* = 0.06) than in congruent sets (*M* = 0.01, *SE* = 0.05). Pairwise comparisons revealed that participants gave more conservative responses in the masked-full condition than in any other condition (cf. Table 2), *t*s(69) *≥* −3.85, *p*s ≤ .001, *d*s* ≥* 0.53. There were no significant differences among the remaining three conditions, *t*s(69) ≤ 0.73, *p*s* ≥* .466, *d*s ≤ 0.10. Furthermore, all of these three conditions displayed unbiased responses, *t*s(69) ≤ 0.74, *p*s ≥ .464, *d*s ≤ 0.09. These findings align with Experiments 1 and 2, demonstrating that contextual congruency can provide gains even when memory is not overly strained.

#### Post-decision confidence

In line with our expectations about the effects of memory load, were able to find support for our Hypothesis 2. The repeated-measures ANOVA on confidence, with contextual congruency and accuracy as factors, yielded a significant main effect of congruency, *F*(1, 61) = 84.68, *p* < .001, *η*_*p*_^*2*^ = .58, and accuracy, *F*(1, 61) = 67.04, *p* < .001,* η*_*p*_^*2*^ = .52. The main effects were qualified by the significant interaction between congruency and accuracy, *F*(1, 61) = 13.95 *p* < .001, *η*_*p*_^*2*^ = .19. Simple-effects analysis showed that in congruent sets, participants were more confident in accurate trials (*M* = 67.90, *SE* = 1.74) than in inaccurate trials (*M* = 57.38, *SE* = 2.01), *t*(63) = 7.14, *p* < .001, *d* = 0.70. Similarly, in incongruent sets, accurate trials (*M* = 52.27, *SE* = 2.06) elicited higher confidence than inaccurate trials (*M* = 48.27, *SE* =2.20), *t*(67) = 4.41, *p* < .001, *d* = 0.23. This effect was noticeably stronger in congruent than incongruent sets as expected (Hypothesis 3). The confidence-accuracy relationship for both congruent and incongruent sets was also supported by the CAC and ROC analyses (cf. Online Supplementary Material). Pairwise comparison revealed that participants rated higher confidence in the masked-partial condition than the two incongruent conditions (full-partial and masked-full), *t*s(69) *≥* 3.04, *p*s ≤ .003, *d*s* ≥* 0.24 (cf. Table 3 and Online Supplementary Material), aligning with Hypothesis 2.

#### Decision time

The repeated-measures ANOVA on decision time, with contextual congruency and accuracy as factors, showed a main effect of congruency, *F*(1, 62) = 9.27, *p* = .003, *η*_*p*_^*2*^ = .13. Supporting Hypothesis 4, participants responded faster in congruent (*M* = 2.88, *SE* = 1.05) than incongruent sets (*M* = 3.16, *SE* = 1.07). Likewise, the main effect of accuracy was significant, *F*(1, 62) = 13.02, *p* < .001, *η*_*p*_^*2*^ = .17, with participants responding faster when making accurate (*M* = 2.88, *SE* = 1.05) than inaccurate decisions (*M* = 3.16, *SE* = 1.07). However, in contrast to Hypothesis 5, the interaction effect between the contextual congruency and accuracy was not significant, *F*(1, 62) = 3.44, *p* = .069, *η*_*p*_^*2*^ = .05. For the pairwise comparisons, with the exception of consistently slower decision time for the masked-full compared to all other conditions, *ts*(69) ≥ −4.69 *ps* < .001, *ds ≥* 0.44, no other systematic differences emerged (cf. Table 3 and Online Supplementary Material). In summary, we found evidence for a congruency effect in decision time when memory load was low but no support for a decision time-accuracy relationship in masked face recognition.

#### Individual differences in masked face recognition

Turning to the relationship between participants’ general recognition ability and recognition performance, the mean CFMT performance in Experiment 3 was *M* = 77.08% (*SD* = 15.06%, range: 42–99%). This translates to a CFMT score of *M* = 55.50 (*SD* = 10.85, range: 30–71). The correlation between participants’ CFMT performance and overall recognition accuracy (see Fig. 2, E3) was moderate and significant, *r*(70) = .42, *p* < .001, 95% CI [.20, .59]. This is in line with Hypothesis 6 and Experiment 2.

Then, we explored the effect of contextual congruency on the relationship between general recognition ability and recognition accuracy (Fig. 3, E3). In the congruent sets, the correlation between CFMT and recognition accuracy was significant and moderate, *r*(70) = .36, *p* = .002, 95% CI [.14, .55]. For the incongruent sets, the correlation between CFMT performance and recognition accuracy was significant but small, *r*(70) = .27, *p* = .026, 95% CI [.03, .47]. Lastly, for all but the full-partial condition the correlations between CFMT performance and each face condition were significant and positive (see Online Supplementary Material).

#### Differences between high- and low-ability performers

Figure 4 illustrates the performance of high- and low-ability performers in congruent and incongruent sets. The mean performance of high-ability performers (*n* = 6) was 96.76% (*SD* = 1.43%, range: 96–99%). This translates to a CFMT score of *M* = 69.67 (S*D* = 1.03, range: 69–71). The mean performance of low-ability performers (*n* = 3) was 42.13% (*SD* = 0.80%, range: 42–43%). This translates to a CFMT score of *M* = 30.33 (*SD* = 0.58, range: 30–31). Our planned repeated-measures ANOVAs on recognition accuracy with contextual congruency and performance as factors revealed no significant main effects, all *F*s(1, 7) ≤ 1.41, *p*s ≥ .273, *η*_*p*_^*2*^ ≤ .17, BF_10_ ≤ .98, or an interaction effect, *F*(1, 7) = 0.08, *p* = .792, *η*_*p*_^*2*^ = .01, BF_10_ = 0.54. The Bayes factors confirmed this impression.

Next, and similar to Experiment 2, we extended the cutoff to include the top and bottom 25% of performers (see Fig. 5). The mean performance of the top 25% performers (*n* = 18) was 93.52% (*SD* = 2.85%, range: 90–99%). This translates to a CFMT score of *M* = 67.33 (*SD* = 2.06; range: 65–71. The mean performance of the bottom 25% performers (*n* = 17) was 55.56% (*SD* = 7.75%, range: 42–64%). This translates to a CFMT score of *M* = 40.00 (*SD* = 5.58, range: 30–46). Similar to Experiment 2, when we extended the cut-off scores we found that congruency, *F*(1, 33) = 21.62, *p* < .001, *η*_*p*_^*2*^ = .40, BF_10_ = 1574.66, and CFMT performance, *F*(1, 33) = 11.25, *p* = .002, *η*_*p*_^*2*^ = .25, BF_10_ = 5.70, had an effect on accuracy. Participants performed better in congruent (*M* = 71.90%, *SE* = 1.90%) than incongruent (*M* = 60.10%, *SE* = 1.90%) sets and high-ability performers (*M* = 70.70%, *SE* = 2.00%) performed better than low-ability performers (*M* = 61.30%, *SE* = 2.00%). However, there was no support for an interaction effect between contextual congruency and CFMT performance, *F*(1, 33) = 0.31, *p* = .583, *η*_*p*_^*2*^ = .01, BF_10_ = 0.36. This suggests that the effects of congruency and CFMT performance on accuracy are independent of each other.

### Discussion

Consistent with our hypotheses and Experiments 1 and 2, participants demonstrated significantly higher sensitivity for congruent compared to incongruent face sets. Importantly, the masked-partial condition outperformed the full-partial condition, aligning with previous findings. In terms of response bias, participants were more conservative in incongruent compared to congruent sets, with the masked-full condition eliciting the most conservative responses. This pattern aligns with Experiments 1 and 2, demonstrating the robust influence of contextual congruency on recognition performance.

By reducing the memory load, we were able to detect the effects of congruency and accuracy on post-decision confidence, with participants being more confident in accurate trials than in inaccurate trials, particularly in congruent conditions. However, while contextual congruency had an effect of on decision time, with participants responding faster in congruent sets, the expected interaction between congruency and accuracy once again did not emerge.

The analysis of differences between high and low-ability performers revealed distinct patterns in recognition accuracy. High-ability performers demonstrated superior performance compared to low-ability performers, with vast differences in mean performances (96.76% vs. 42.13%). Upon extending the cutoff to include the top and bottom 25% of performers (as opposed to the top and bottom 5% of performers) and increasing power, high-ability performers outperformed low-ability performers and the effects of congruency and accuracy appeared to be independent of each other.

## General discussion

In a line of three experiments, we explored the beneficial value of congruency and feature-based processing for improving face recognition performance for masked perpetrators. Additionally, we investigated the potential influence of varying levels of face recognition ability on recognition performance in masked face recognition. The results consistently supported the preregistered hypothesis that face recognition is improved when there is congruency, rather than incongruency, between the encoding and retrieval phases (Hypothesis 1). When encoding masked faces, participants were better at recognizing partial faces than full faces, with recognition of masked targets benefiting from limiting the facial features available on the face to be recognized (cf. Table 2). Regarding general face recognition ability, we found a moderate correlation between participants' general ability to recognize faces and recognition accuracy for masked targets under conditions of contextual congruency. Furthermore, we found that limits in face memory load can affect the relationship between confidence and accuracy (see Experiment 3).

We consistently found that discriminability was significantly higher in congruent than incongruent sets (also see recognition accuracy and ROC curves in the Online Supplementary Material).These findings align with previous research showing that contextual congruency plays a key role in facilitating matching performance for masked perpetrators (Estudillo & Bindemann, 2014; Guerra et al., 2022; Or et al., 2023; Zhang et al., 2023). Notably, our work extends these findings to the recognition of faces under severe constraints on the facial features, where only limited facial features are visible (cf. Fig. 1), further emphasizing the importance of congruency, especially for masked faces. When participants encoded masked faces, they consistently performed better at recognizing partial than full faces. These findings are interpreted within the conceptual framework of feature-based processing, emphasizing its role in understanding face recognition for masked targets. They align with the idea that focusing on specific facial features can improve masked face recognition (Bonner et al., 2003; Burton et al., 2010; Megreya & Burton, 2006). However, while the results are interpreted through the lens of feature-based processing, it is important to note that the study was not explicitly designed to test this theoretical approach.

Response bias was also influenced by the congruency between the encoding and retrieval phases in all three experiments. Overall, participants tended to have a conservative response bias in incongruent masked sets. However, the picture gets more nuanced when looking at the encoded face. When encoding a full face, participants exhibited a neutral or conservative bias (see Table 2), regardless of retrieval type. Conversely, when encoding a masked face, participants exhibited a liberal response bias when retrieving a partial face and a strong conservative response bias when retrieving a full face. These findings support the notion that masks can shift people's decisions from neutral to biased (Or et al., 2023; Verde & Rotello, 2007) and further demonstrate that retrieval with different contexts in masked face recognition can also affect decision criteria (Garcia-Marques et al., 2022; Guerra et al., 2022).

Turning to individual differences in masked face recognition, we found support for the hypothesis that general face recognition ability would correlate positively with recognition performance in both congruent and incongruent conditions (Hypothesis 6). While we found significant positive correlations between general face recognition ability and recognition accuracy for masked targets, the magnitude of the effect differed between congruent and incongruent sets. Congruent sets elicited a moderate effect, whereas incongruent sets elicited a small effect. However, we found no support for the idea that high-ability face performers would maintain the congruency effect, while low-ability performers would perform equally in congruent and incongruent sets. Both high- and low-ability performers demonstrated a congruency effect but the effect for low-ability performers was weaker in Experiment 2. Taken together, maintaining high levels of recognition ability across conditions was a difficult task for our participants. This is consistent with previous work showing that while some people with exceptional recognition ability are very good at matching faces, they are not necessarily equally good at all types of tasks (Bobak et al. 2016). At a theoretical level, these findings provide moderate support for the notion that people with superior face recognition abilities make more effective use of visible features to activate their stored memory representations irrespective of the processing strategy used (Dunn et al., 2022; Royer et al., 2018).

For postdictors of accuracy, in Experiments 1 and 2, we found no congruency effect in confidence ratings and decision times (Hypothesis 2 and Hypothesis 4), and no relationship between confidence and accuracy and between decision time and accuracy (Hypothesis 3 and 5). The lack of a contextual congruency effect in confidence and response time is consistent with earlier findings in a matching task with masked faces (Zhang et al., 2023), but contradicts lineup studies (Dunning & Perretta, 2002; Sauerland & Sporer, 2009; Sauerland et al., 2018; Sousa & Jaeger, 2022; Sporer et al., 1995; Wixted & Wells, 2017). The lack of a confidence-accuracy and a decision time-accuracy relationship could be explained by task difficulty. Individuals assess the contents and the memory strength of the stored items to make confidence judgments (Busey et al., 2000; Hart, 1967). This can be particularly challenging when many faces have to be remembered, making it difficult to align confidence ratings with accuracy (Kramer et al., 2022; Lichtenstein & Fischhoff, 1977). Similarly, face masks impair the holistic processing of a face (Carragher & Hancock, 2020; Freud et al., 2020). This could potentially result in individuals needing to spend more time extracting consistent feature representations from memory for masked faces (Craik et al., 2000). The more faces are stored, the higher the face load that participants experience.

Indeed, when we reduced the number of faces that participants had to memorize (Experiment 3), we found a congruency effect on confidence (Hypothesis 2) and a clear confidence-accuracy relationship (Hypothesis 3), for positive decisions (as evidenced by the CAC analysis shown in Online Supplementary Material). These findings are consistent with previous studies including lineup studies (Brewer & Wells, 2006; Dodson & Dobolyi, 2016; Gettleman et al., 2021; Grabman & Dodson, 2020; Weber et al., 2013; Weber & Brewer, 2006; Weber et al., 2013). However, despite the congruency effect on decision times (Hypothesis 4), we did not find the expected decision time-accuracy relationship. Together, these findings suggest that memory load has an impact on both the congruency effect and the confidence-accuracy relationship and support the notion of a trade-off between processing and storage (Lamont & Stewart-Williams, 2005; Lavie et al., 2003; Podd, 1990). Finally, the results cautiously suggest that decision time may not be a reliable indicator of accuracy for masked targets.

## Conclusion

There is an ongoing debate about whether the accuracy of recognizing masked individuals can be improved by making the context of the faces being compared more consistent, known as contextual congruency. The current line of research shows that congruency between encoding and retrieval is important for recognizing masked targets. In addition, people with high face recognition ability may not be able to maintain their high performance across retrieval conditions. This suggest that law enforcement agencies may benefit from providing witnesses with partial features rather than the full face of a suspect when a perpetrator is masked. However, they should be cautious in assuming that people who are generally good at recognizing faces would outperform less able counterparts in situations involving masked targets. From a theoretical point of view, our work suggests that limitations in memory load may also affect the relationship between confidence and accuracy and, in turn, the role of confidence as a predictor of accuracy. Moving forward, it would be valuable to explore how these findings apply in real-world scenarios, where perpetrators wearing masks are often captured in lower-quality images, such as those from CCTV footage. Investigating how these variations in image quality affect recognition accuracy and contextual congruency will help assess the broader applicability of the study's conclusions.

## Supplementary information

Below is the link to the electronic supplementary material.

Supplementary file1 (DOCX 850 KB)

## Data Availability

The datasets generated and analyzed in the current study are available in the Dataverse repository [https://dataverse.nl/privateurl.xhtml?token=50ec6396-8f24-4827-8085-c0f32ad58d30].

## Footnotes

1 The design did not incorporate gender into the analysis as pre-registered. Due to the main text focusing on the role of congruency effects in masked face recognition, we report the exploratory analysis of gender in the Online Supplementary Material.

2 We use the following formulas: *d'* = z(hit) - z(false alarm),and c = −[z(hit) + z(false alarm)]/2. In cases where participants' responses were either 100% accurate or inaccurate we applied the 1/(2*N*) correction rule (Macmillan & Creelman, 2005).

3 The differences in the degrees of freedom in the analysis of confidence and decision time are due to some participants (*n* = 8) in Experiment 3 answering correctly on all trials. This resulted in no incorrect trials and consequently missing values for these participants on these measures, explaining the reduced degrees of freedom.

## Code availability

Custom code used in the analysis of the data is available from the corresponding author upon request.
