# Supplementary material for: Congruency effects and individual differences in masked face recognition under limited feature visibility
Source: Mem Cognit. 2025 Mar 12;53(7):2076–93. doi: 10.3758/s13421-025-01699-9 (PMC12589217; doi:10.3758/s13421-025-01699-9)
Supplement: Supplementary file 1 — Supplementary file1 (DOCX 850 KB) [file 13421_2025_1699_MOESM1_ESM.docx]

**Supplementary**

**Face Recognition Performance**

In line with Hypothesis 1, the main effect of contextual congruency on accuracy was significant in Experiment 1, *F*(1, 131) = 269.34, *p* < .001, *η_p_^2^* = .67, Experiment 2, *F*(1, 113) = 132.96, *p* < .001, *η_p_^2^* = .54, and Experiment 3, *F*(1, 69) = 62.71, *p* < .001, *η_p_^2^* = .48. And all of these three experiments showed a large effect size. Recognition accuracy was better in the congruent trials (E1: *M* = .67, *SE* = 0.01; E2: *M* = .66, *SE* = .01; E3: *M* = .66, *SE* = .01) than incongruent trials (E1: *M* = .55, *SE* = .01; E2: *M* = .55, *SE* = .01; E3: *M* = .55, *SE* = .01). Thus, all experiments showed the congruency effect in recognition performance, with higher performance in congruent sets than incongruent sets in masked face recognition.

To examine potential differences between the four conditions (full-full, masked-partial, full-partial, and masked-full), we used paired samples t‐tests. Table S1 shows the descriptive statistics. As expected, participants performed significantly better in the full-full condition than in any other condition in three experiments (E1: all *ts*(131) *≥* 11.52, *ps* < .001, *ds* *≥* 1.27; E2: all *ts*(113) *≥* 10.06, *ps* < .001, *ds* *≥* 1.12; E3: *ts*(69) *≥* 6.86, *ps* < .001, *ds* *≥* 1.03). Also as expected, the masked-partial condition outperformed both the masked-full, (E1: *t*(131) = 3.54, *p* < .001, *d* = 0.40; E2: *t*(113) = 2.97, *p* = .004, *d* = 0.32; E3: *t*(69) = 2.32, *p* = .023, *d* = 0.39), and the full-partial condition, (E1: *t*(131) = -5.09, *p* < .001, *d* = 0.60; E2: *t*(113) = -4.74, *p* = .004, *d* = 0.60; E3: *t*(69) = -2.99, *p* = .004, *d* = 0.57). Last, the comparison between incongruent sets did not differ significantly in Experiment 1 and 3 (E1: *t*(131) = -1.54, *p* = .125, *d* = 0.20; E3: *t*(69) = -0.91, *p* = .368, *d* = 0.14). However, in Experiment 2, participants performed better in the full-partial condition than the masked-full condition, *t*(113) = 2.50, *p* = .014, *d* = 0.30. This finding aligned with Hypothesis 1, indicating that participants demonstrated superior performance in the masked congruent sets compared to the masked incongruent sets. This suggests that recognising features, rather than full faces, can enhance masked face recognition performance.

**Table S1**

*Descriptive Statistics for Face Recognition Accuracy for Congruent and Incongruent Sets in Three Experiments*

|  |  | *Congruent* | |  | *Incongruent* | |
| --- | --- | --- | --- | --- | --- | --- |
|  |  | *Full-full*^a^ | *Masked-partial*^b^ |  | *Full-partial*^c^ | *Masked-full^d^* |
| Experiment 1 | *M (SD)* | 0.74 (0.12) | 0.60 (0.10)^a***^ |  | 0.54 (0.10)^ab***^ | 0.56 (0.10)^ab***^ |
|  | *95% CI* | [0.72, 0.76] | [0.59, 0.61] |  | [0.53, 0.55] | [0.55, 0.57] |
| Experiment 2 | *M (SD)* | 0.72 (0.13) | 0.59 (0.10)^a***^ |  | 0.53 (0.10)^ab***^ | 0.56 (0.09)^a***b**c**^ |
|  | *95% CI* | [0.70, 0.74] | [0.58, 0.61] |  | [0.52, 0.55] | [0.55, 0.57] |
| Experiment 3 | *M (SD)* | 0.80 (0.14) | 0.65 (0.15)^a***^ |  | 0.57 (0.13)^a***b**^ | 0.59 (0.27)^a***b*^ |
|  | *95% CI* | [0.77, 0.83] | [0.62, 0.68] |  | [0.54, 0.60] | [0.56, 0.62] |

*Note.* The superscript letters indicate significant differences from ^a^ = full-full, ^b^ = masked-partial, ^c^ = full-partial, ^d^ = masked-full, at ^*^*p* < .05, ^**^*p* < .01, ^***^ *p* < .001.

**Post-Decision Confidence**

Table 3 in the main text displays the mean post-decision confidence ratings for each condition in each Experiment. In Experiment 1 and 2, decisions in the full-full condition were made with similar confidence as those in the full-partial condition (E1: *t*(131) = 0.37, *p* = .715, *d* = 0.01; E2: *t*(113) = .23, *p* = .816, *d* < 0.01), but with higher confidence than in any other condition (E1: *ts*(131) *≥* 5.68, *ps* < .001, *ds* *≥* 0.33. E2: *ts*(113) *≥* 3.48, *ps* < .001, *ds* *≥* 0.21). Surprisingly, confidence did not differ between the masked-partial and the masked-full conditions (E1: *t*(131) = 0.47, *p* = .638, *d* = 0.02, E2: *t*(113) = -0.09, *p* = .925, *d* < 0.01), but confidence in the masked-partial condition was significantly lower than the full-partial condition (E1: *t*(131) = 6.31, *p* < .001, *d* = 0.33; E2: *t*(113) = 3.45, *p* < .001, *d* = 0.20). The difference between the two incongruent trials (full-partial vs. masked full) was significant (E1: *t*(131) = 5.56, *p* < .001, *d* = 0.31; E2: *t*(113) = 3.37, *p* < .001, *d* = 0.20). These findings are not fully in line with our expectations (Hypothesis 2).

Experiment 3 investigated the potential impact of cognitive memory overload on confidence. Unlike the previous two experiments, the results of Experiment 3 showed that participants rated significantly higher confidence in the full-full condition than for any other conditions, *ts*(69) *≥* 8.64, *ps* ≤ .001, *ds ≥* 1.03. Likewise, participants rated higher confidence in the masked-partial condition than in the masked-full condition, *t*(69) = 3.04, *p* = .003, *d* = 0.24, and in the full-partial condition, *t*(69) = 7.41, *p* < .001, *d* = 0.56. Last, confidence was higher for the masked-full condition than the full-partial condition, *t*(69) = 3.79, *p* < .001, *d* = 0.32. These results in line with Hypothesis 2, participants exhibited heightened confidence in recognizing congruent sets with masked stimuli compared to incongruent sets.

**ROC Curves**

To examine whether a stronger confidence-accuracy relationship in congruent than incongruent trials (Hypothesis 3), we conducted ROC analyses using the ROC toolbox (Koen et al., 2017) to fit the unequal-variance signal detection (UVSD) model (Mickes et al., 2012). This model examines the response frequencies for correct and incorrect answers separately for responses to “old” and “new” faces. To determine response bias and discriminability at different confidence levels, we divided the confidence ratings into 20 bins in descending order. We established Area Under the Curve (AUC) to compare the accuracy of different conditions, with higher AUC values indicating better performance. We used the Delong test (Delong et al., 1988) to test for statistically significant differences between AUCs.

Figure S1 shows the confidence-based ROC curves for congruent and incongruent trials. The more the plotted points diverge up and away from the diagonal line of chance performance, the better people can distinguish “old” faces from “new” faces across distinct levels of confidence. The results consistently demonstrate that discriminability was significantly higher for congruent (E1: AUC = .69; E2: AUC = .67; E3: AUC = .78) than incongruent trials (E1: AUC = .53; E2: AUC = .55; E3: AUC = .54), *ps* < .001. These findings support the idea of a congruency effect. In brief, participants displayed better discriminability in the congruent trials than in incongruent trials across all experiments.

**Figure S1**

*Confidence Based ROC Curves for Congruent and Incongruent Face Sets for Experiment 1 (E1), Experiment 2 (E2) and Experiment 3 (E3)*

**
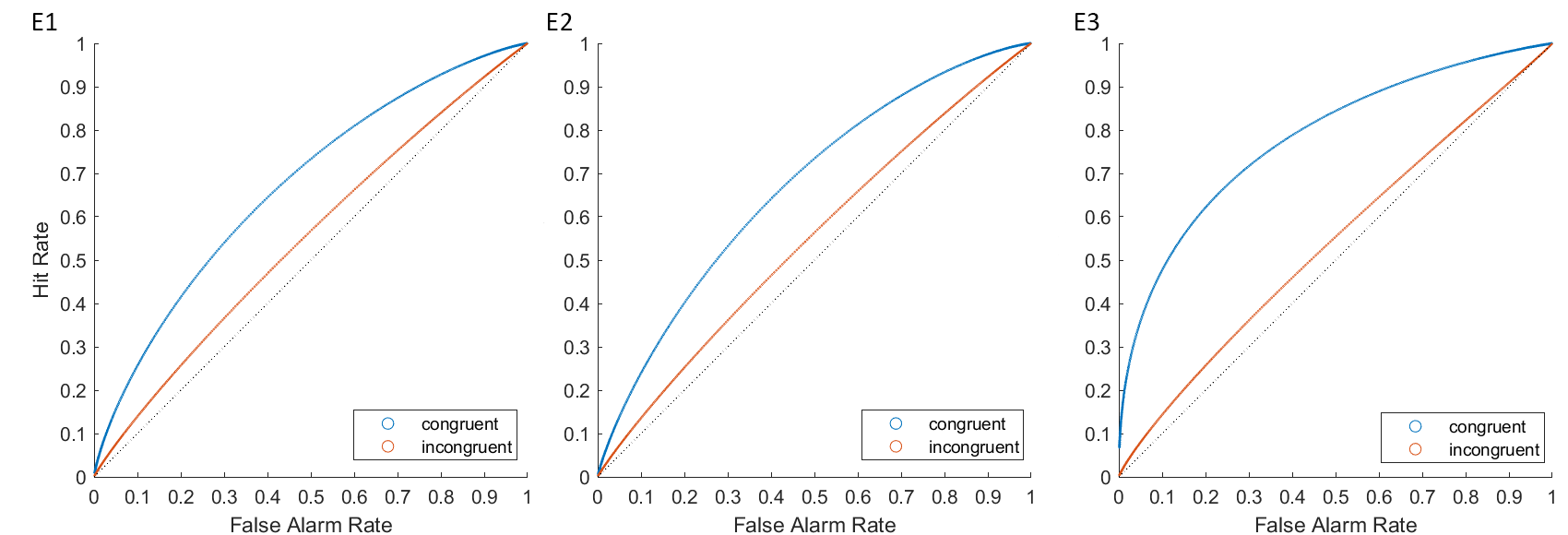
***Note.* The panel shows the ROC curves for congruent (full-full and masked-partial) versus incongruent (full-partial and masked-full) trials. The diagonal line represents chance performance.

Comparing the four conditions (see Figure S2), the full-full condition (E1: AUC = .75; E2: AUC = .74; E3: AUC = .85) yielded the highest discriminability, compared to all other conditions (*ps* < .001). Likewise, discriminability in the masked-partial condition (E1: AUC = .60; E2: AUC = .61; E3: AUC = .68) was significantly higher than in the masked-full condition (E1: AUC = .56, *p* = .022; E2: AUC = .55, *p* < .001; E3: AUC = .59, *p* < .001), and in the full-partial condition (E1: AUC = .55, *p* = .004; E2: AUC = .54, *p* < .001; E3: AUC = .50; *p* < .001). The difference between the two incongruent sets was non-significant (E1: *p* = .571; E2: *p* = .572). However, the difference between the two incongruent conditions in Experiment 3 was significant, *p* < .001.

**Figure S2**

*Confidence Based ROC Curves for Each Experimental Condition for Experiment 1 (E1), Experiment 2 (E2) and Experiment 3 (E3)*


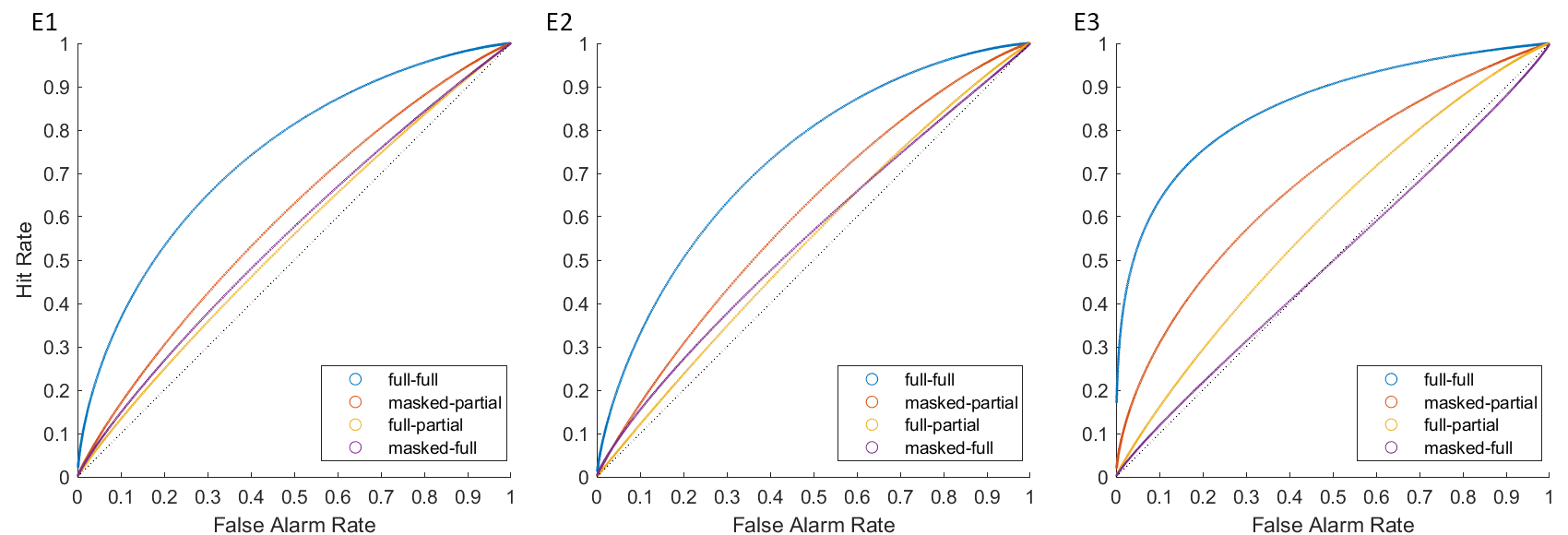
*Note.* The panel shows the ROC curves separately for each experimental condition. The diagonal line represents chance performance.

These results further corroborate the findings of the signal detection analyses, indicating that participants demonstrated superior ability to discriminate faces in congruent trials compared to incongruent sets (Hypothesis 1) across all experiments. Notably, when encoding masked faces, participants exhibited enhanced discrimination of partial faces compared to full faces. This suggests that facial features can be a valuable cue for recognizing masked faces.

**Confidence-Accuracy Characteristics (CAC) Curves**

To examine whether a stronger confidence-accuracy relationship in congruent than incongruent sets (Hypothesis 3), we conducted the confidence accuracy characteristic (CAC) curves. The CAC curves plotted the proportion of correct recognition across six bins of confidence (up to 50%, 51%-60%, 61%-70%, 71%-80%, 81%-90%, 91%-100%; for a similar approach see Stephens et al., 2017). Figure S3 shows the curves for participants who made positive (“old”, have seen the face before) and negative decisions (“new”, never seen the face before) for all three experiments. The slopes for Experiment 1 and 2 are quite flat, indicating poor calibration between confidence and accuracy for both positive and negative decisions. In Experiment 3, the CAC curve plots show a clear confidence accuracy relationship for positive decisions, consistent with Hypothesis 3. For negative decisions, the slopes were again quite flat, except for the full-partial condition, for which the curve suggests a negative relationship between confidence and accuracy.

**Figure S3**

*Confidence–Accuracy Characteristic (CAC) Plots for Positive (“Have seen this face before**”, upper Panel) Decisions and Negative (“Never seen this face before**”, lower Panel) Decisions for Each Condition in Experiment 1 (E1), Experiment 2 (E2), and Experiment 3 (E3)*

**
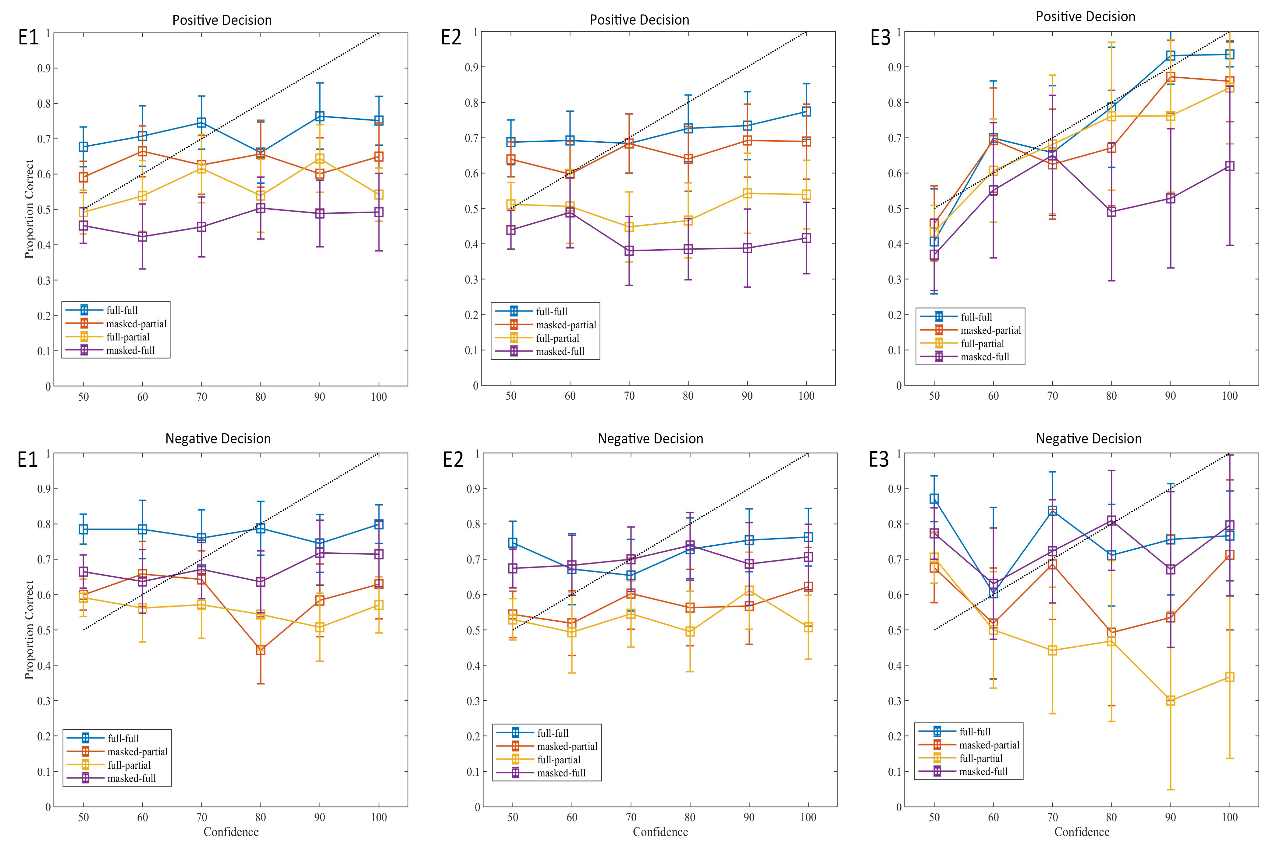
**

*Note.* The upper panel shows the CAC for participants who made positive decisions, and the lower panel shows the CAC for participants who made negative decisions. Error bars show 95% confidence intervals for the proportions, the dotted line is the calibration line.

**Decision Time**

Table 3 in the main text displays the mean decision times for each condition in each Experiment. In Experiment 1 and 2, inconsistent with Hypothesis 4, decision times in the full-full condition were similar to those in the full-partial condition (E1: *t*(131) = 0.65, *p* = .517, *d* = 0.02; E2: *t*(113) = 0.35, *p* = .730, *d* = 0.01), but faster than in the other conditions (E1: *ts*(131) *≥* 5.68, *ps* < .001, *ds* *≥* 0.33; E2: *ts*(113) *≥* 6.36, *ps* < .001, *ds* *≥* 0.33). Unexpectedly, decision times did not differ between the masked-partial and the masked-full condition (E1: *t*(131) = 0.65, *p* = .518, *d* = 0.02; E2: *t*(113) = 0.85, *p* = .395, *d* = 0.03), but participants responded significantly slower in the masked-partial condition than the full-partial condition (E1: *t*(131) = 5.68, *p* < .001, *d* = 0.26; E2: *t*(113) = 6.65, *p* < .001, *d* = 0.38). The two incongruent conditions (full-partial vs. masked-full) differed significantly (E1: *t*(131) = 5.12, *p* < .001, *d* = 0.26; E2: *t*(113) = 6.55, *p* < .001, *d* = 0.36), with slower decision times in the masked-full condition than the full-partial condition.

In Experiment 3, participants responded faster in the full-full condition than the masked-full condition, *t*(69) = 5.91, *p* < .001, *d* = 0.48, but there was no difference for any other condition, *ts*(69) ≤ 1.16, *ps ≥* .249, *ds* ≤ 0.11. Likewise, participants responded faster in the masked-partial condition than the masked-full condition, *t*(69) = 8.23, *p* < .001, *d* = 0.61, but there was no difference between the masked-partial condition and the full-partial condition, *t*(69) = 1.75, *p* = .085, *d* = 0.17. Last, decisions were made faster in the full-partial condition than the masked-full condition, *t*(69) = 4.69, *p* < .001, *d* = 0.44.

**Individual Differences in Masked Face Recognition**

As part of the exploratory analysis related to Hypothesis 6, we explored the relationship between participants’ general recognition ability and recognition performance for masked targets. Specifically, we examined the correlation between participants’ CFMT performance and recognition accuracy of each condition in Experiments 2 and 3, see Figure S4 and S5. The full-full condition generated moderate and significant correlation coefficients in both experiments (E2: *r =* .36, *p* < .001, *95% CIs* = [.19, .51]; E3: *r =* .30, *p* = .011, *95% CIs* = [.07, .50]). The masked-partial condition also generated moderate and significant correlation coefficients (E2: *r =* .40, *p* < .001, *95% CIs* = [.23, .54]; E3: *r =* .28, *p* = .002, *95% CIs* = [.04, .48]). The differences between these two conditions was not significant (E2: *p* = .728; E3: *p* = .899). For the incongruent conditions, the relationship between CFMT performance and recognition accuracy in the full-partial condition was non-significant in both experiments (E2: *r =* .14, *p* = .145, *95% CIs* = [-.05, .31]; E3: *r =* .16, *p* = .175, *95% CIs* = [-.07, .39]). For the masked-full condition, the relationship between CFMT performance and recognition accuracy was significant but small (E2: *r =* .24, *p* = .009, *95% CIs* = [.06, .41]; E3: *r =* .24, *p* = .047, *95% CIs* = [.00, .45]). The difference between the two congruent conditions was not significant (E2: *p* = .439; E3: *p* = .629).

**Figure S4**

*The Correlations between Different Conditions of Face Recognition Performance and CFMT in Experiment 2.*


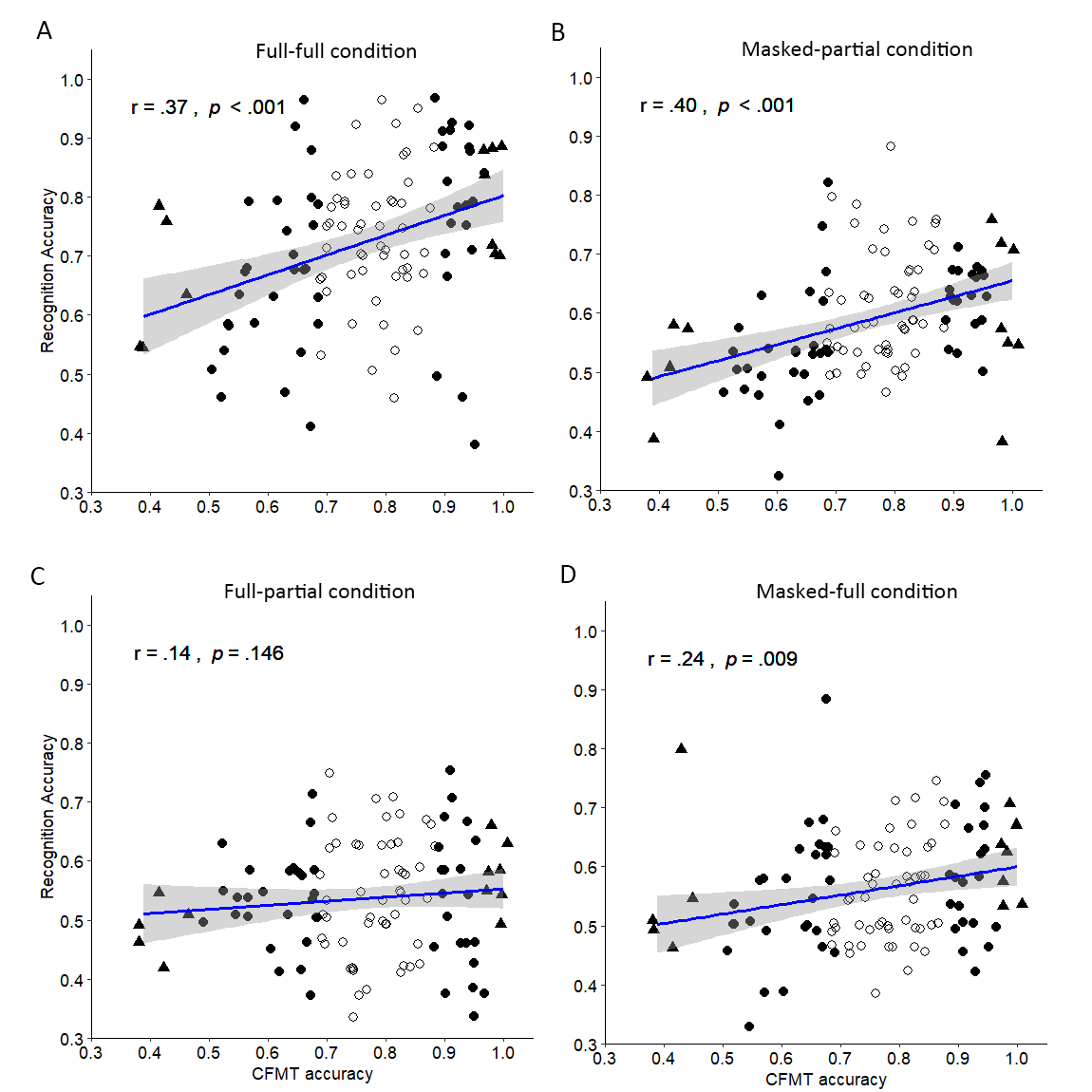


*Note.* Scatterplots show the relationship between the Cambridge Face Memory Test (CFMT) and face recognition performance for (A) full-full condition, (B) masked-partial condition, (C) full-partial condition and (D) masked-full condition. Participants with the top 5% and the bottom 5% scores in the CFMT are presented by triangles; the top 25% performers and bottom 25% performers are presented by solid circles, 95% CI in grey.

**Figure S5**

*Correlations Between Face Recognition Performance and the CFMT for Four Different Conditions in Experiment 3.*

*
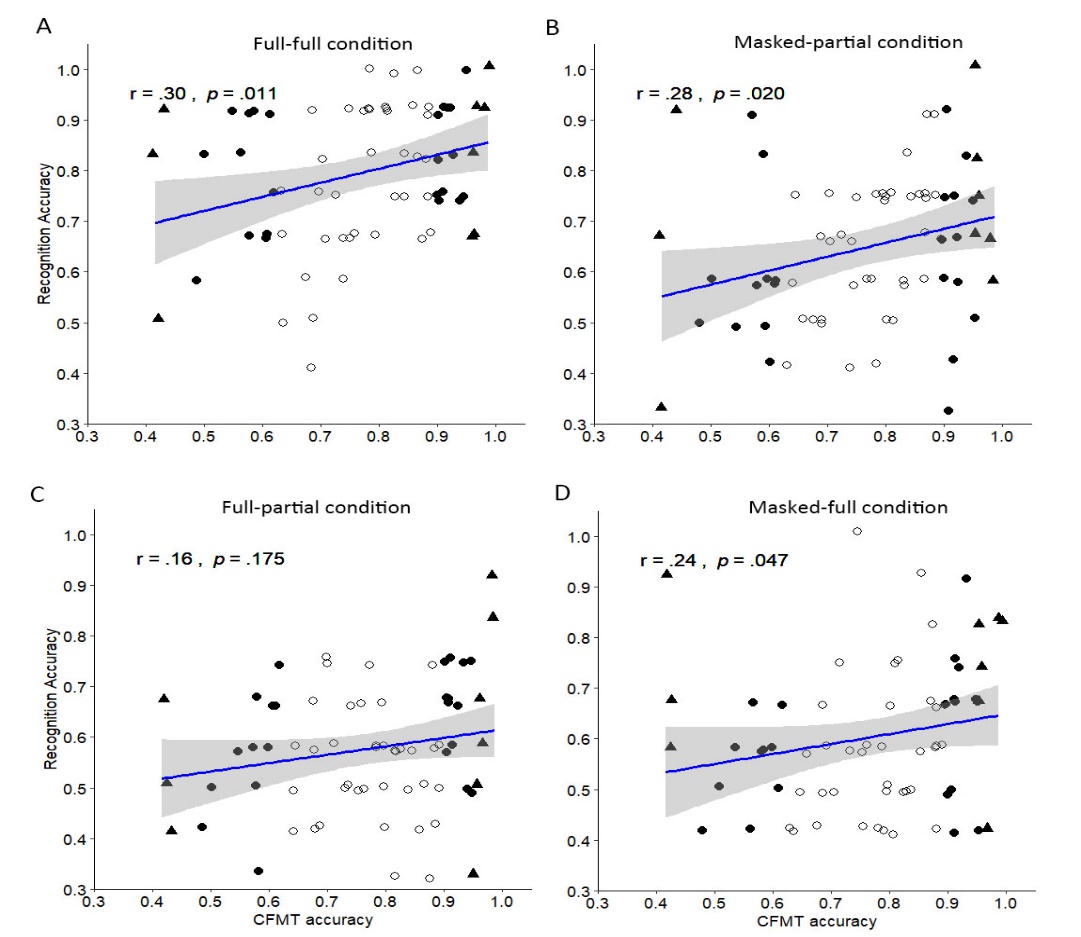
*

*Note.* Scatterplots show the relationship between the Cambridge Face Memory Test (CFMT) and face recognition performance for (A) full-full condition, (B) masked-partial condition, (C) full-partial condition and (D) masked-full condition. Participants with the top 5% and the bottom 5% scores in the CFMT are presented by triangles; the top 25% performers and bottom 25% performers are presented by solid circles, 95% CI in grey.

**Memory Load**

To explore whether the memory load impacted the congruency effect in masked face recognition among Experiment 1, 2 and 3, we conducted a mixed ANOVA testing the effect of memory load (high/Experiment 1 and 2 vs. low/Experiment3) and congruency (congruent vs. incongruent) on sensitivity. The main effect of congruency was significant, *F*(1, 314) = 268.86, *p* < .001, *η_p_^2^* = .46, indicating higher sensitivity for congruent (*M* = 1.23, *SE* = .05) than incongruent trials (*M* = 0.42, *SE* = 0.04) in all experiments. Likewise, the main effect of memory load was also significant, *F*(1, 314) = 15.37, *p* < .001, *η_p_^2^* = .05. Participants displayed higher sensitivity under the low memory load in Experiment 3 (*M* = 0.95, *SE* = 0.06) than the high memory load in Experiment 1 and 2 (*M* = 0.69, *SE* = 0.03). The interaction was non-significant, *F*(1, 314) = 1.09, *p* = .297, *η_p_^2^* < .01. This suggests that the congruency effect was consistent, regardless of memory load.

**Gender Effect**

In addition to the hypothesis-driven analyses, we preregistered and conducted an exploratory investigation into how masks influence gender bias in the recognition of masked perpetrators. Previous research has documented the own-gender bias, where individuals exhibit better memory for faces of their own gender compared to the opposite gender (Sporer, 2001; Herlitz & Lovén, 2013). This phenomenon has been extensively studied in full-face views (Herlitz & Lovén, 2013; Rehnman & Herlitz, 2007; Wong & Estudillo, 2022), but the impact of masks on gender bias remains under-explored.

Recent studies have indicated that masks can impair gender classification, reducing accuracy by nearly 5% (Wong and Estudillo, 2022). This suggests that masks significantly affect the ability to judge the gender of a masked face. However, another study found no significant differences between male and female participants when matching masked male and female perpetrators, indicating that the own-gender bias may not be supported when perceiving masked targets (Zhang et al., 2023). Given that face recognition involves both perceptual and memory processes, we wanted to further elucidate the potential impact of gender bias on the recognition of masked faces.

We preregistered and conducted a mixed ANOVA with accuracy as the outcome variable and participant gender (male vs. female), target gender (male vs. female), and contextual congruency (congruent vs. incongruent) as factors. The the following, we only report the main effects of participant gender, target gender, and their interactions with congruency.

Experiment 1 and 2 revealed a significant main effect of participant gender (E1: *F*(1, 130) = 13.29, *p* < .001, *η_p_^2^* = 0.09; E2: *F*(1, 112) = 4.48, *p* = .037, *η_p_^2^* = .04). Female participants were more accurate than male participants. The main effect of target gender was significant in Experiment 1, *F*(1, 130) = 4.12, *p* = .045, *η_p_^2^* = 0.03, but not in Experiment 2, *F*(1, 112) = 1.09, *p* = .299, *η_p_^2^* = .01. In Experiment 1, male participants and female participants were more accurate when seeing the female target than the male target. As for the interaction effects, we only found the significant congruency by target gender interaction in Experiment 2, *F*(1, 112) = 18.11, *p* < .001, *η_p_^2^* = .14. Simple main effects revealed lower recognition accuracy for female (*M* = .64, *SE* = .01) than male faces (*M* = .67, *SE* = .01) in congruent trials, *F*(1, 112) = 6.25, *p* = .014, *η_p_^2^* = .05, but higher accuracy for female (*M* = .57, *SE* = .01) than male faces (*M* = .52, *SE* = .01) in incongruent trials, *F*(1, 112) = 13.86, *p* < .001, *η_p_^2^* = .11. All other two-way interactions (E1: *Fs*(1, 130) ≤ 2.25, *ps ≥* .136, *η_p_^2^s* ≤ 0.02; E2: *Fs*(1, 112) ≤ 2.71, *ps ≥* .102, *η_p_^2^s* ≤ 0.02) and the three-way interaction (E1: *F*(1, 130) = 0.33, *p* = .568, *η_p_^2^* < .01; E2: *F*(1, 112) = 2.57, *p* = .112, *η_p_^2^* = .02) were not significant.

In Experiment 3, neither the main effect of target gender, *F*(1, 68) = 0.16, *p* = .689, *η_p_^2^* < .01, nor participant gender was significant, *F*(1, 68) = 0.30, *p* = .584, *η_p_^2^* < .01. Finally, similar to Experiment 1 and 2, neither the two-way interactions, *F*(1, 68) *≤* 3.11, *p* *≥* .082, *η_p_^2^* *≤* 0.04, nor the three-way interaction were significant, *F*(1, 68) = 0.04, *p* = .845, *η_p_^2^* < .01.

Overall, the congruency effect in face recognition demonstrated remarkable stability across all experiments, independent of gender. Both female and male participants exhibited superior performance in congruent compared to incongruent trials, underscoring the impact of encoding-retrieval congruency on face recognition processes. Additionally, consistent with earlier findings (Herlitz & Lovén, 2013; Rehnman & Herlitz, 2006; 2007), female participants in Experiment 1 and 2 were more accurate at recognizing faces than male participants. Despite obscuring most facial features in our experimental paradigm, the female advantage in face recognition persisted. This suggests that limited exposure to the eye region serves as a critical cue in enhancing masked face recognition. However, the gender effect did not emerge in Experiment 3. The reason could be that higher working memory loads may cause performance differences that may not be seen at lower loads (Reed et al., 2017). Nonetheless, these findings underscore that the congruency effect appears robust against gender-related variations.

# References

Delong, E. R., Delong, D. M., Clarke-Pearson, D. L., & Carolina, N. (1988). Comparing the areas under two or more correlated receiver operating characteristic curves: a nonparametric approach. *Biometrics*, *44*(3), 837–845.

Herlitz, A., & Lovén, J. (2013). Sex differences and the own-gender bias in face recognition: A meta-analytic review. *Visual Cognition*, *21*(9–10), 1306–1336. https://doi.org/10.1080/13506285.2013.823140

Mickes, L., Flowe, H. D., & Wixted, J. T. (2012). Receiver operating characteristic analysis of eyewitness memory: Comparing the diagnostic accuracy of simultaneous versus sequential lineups. *Journal of Experimental Psychology: Applied*, *18*(4), 361–376. https://doi.org/10.1037/a0030609

Reed, J. L., Gallagher, N. M., Sullivan, M., Callicott, J. H., & Green, A. E. (2017). Sex differences in verbal working memory performance emerge at very high loads of common neuroimaging tasks. *Brain and Cognition*, *113*, 56–64. https://doi.org/10.1016/j.bandc.2017.01.001

Rehnman, J., & Herlitz, A. (2006). Higher face recognition ability in girls: Magnified by own-sex and own-ethnicity bias. *Memory*, *14*(3), 289–296. https://doi.org/10.1080/09658210500233581

Rehnman, J., & Herlitz, A. (2007). Women remember more faces than men do. *Acta Psychologica*, *124*(3), 344–355. https://doi.org/10.1016/j.actpsy.2006.04.004

Stephens, R. G., Semmler, C., & Sauer, J. D. (2017). The effect of the proportion of mismatching trials and task orientation on the confidence-accuracy relationship in unfamiliar face matching. *Journal of Experimental Psychology: Applied*, *23*(3), 336–353. https://doi.org/10.1037/xap0000130

Wong, H. K., & Estudillo, A. J. (2022). Face masks affect emotion categorisation, age estimation, recognition, and gender classification from faces. *Cognitive Research: Principles and Implications*. https://doi.org/10.1186/s41235-022-00438-x

Zhang, M., Sauerland, M., & Sagana, A. (2023). Improving identity matching for masked faces: The benefit of isolated facial features. *PsyArXiv*, https://doi.org/10.31234/osf.io/j526c
